# Viral ecogenomics across the Porifera

**DOI:** 10.1186/s40168-020-00919-5

**Published:** 2020-10-02

**Authors:** Cecília Pascelli, Patrick W. Laffy, Emmanuelle Botté, Marija Kupresanin, Thomas Rattei, Miguel Lurgi, Timothy Ravasi, Nicole S. Webster

**Affiliations:** 1 AIMS@JCU, Townsville, Queensland, Australia; 2 Australian Institute of Marine Science, PMB No.3, Townsville MC, Townsville, Queensland 4810 Australia; 3 James Cook University, Townsville, Australia; 4 KAUST Environmental Epigenetic Program (KEEP), Division of Biological and Environmental Sciences & Engineering, King Abdullah University of Science and Technology, Thuwal, Kingdom of Saudi Arabia; 5 Department of Microbiology and Ecosystem Science, Division of Computational Systems Biology, University of Vienna, Vienna, Austria; 6 Biosciences Department, University of Swansea, Swansea, Wales; 7 Australian Centre for Ecogenomics, University of Queensland, Brisbane, Australia

**Keywords:** Viromics, Viral ecology, Functional diversity, AMGs, Coral reef sponges

## Abstract

**Background:**

Viruses directly affect the most important biological processes in the ocean via their regulation of prokaryotic and eukaryotic populations. Marine sponges form stable symbiotic partnerships with a wide diversity of microorganisms and this high symbiont complexity makes them an ideal model for studying viral ecology. Here, we used morphological and molecular approaches to illuminate the diversity and function of viruses inhabiting nine sponge species from the Great Barrier Reef and seven from the Red Sea.

**Results:**

Viromic sequencing revealed host-specific and site-specific patterns in the viral assemblages, with all sponge species dominated by the bacteriophage order *Caudovirales* but also containing variable representation from the nucleocytoplasmic large DNA virus families *Mimiviridae*, *Marseilleviridae*, *Phycodnaviridae*, *Ascoviridae*, *Iridoviridae*, *Asfarviridae and Poxviridae*. Whilst core viral functions related to replication, infection and structure were largely consistent across the sponge viromes, functional profiles varied significantly between species and sites largely due to differential representation of putative auxiliary metabolic genes (AMGs) and accessory genes, including those associated with herbicide resistance, heavy metal resistance and nylon degradation. Furthermore, putative AMGs varied with the composition and abundance of the sponge-associated microbiome. For instance, genes associated with antimicrobial activity were enriched in low microbial abundance sponges, genes associated with nitrogen metabolism were enriched in high microbial abundance sponges and genes related to cellulose biosynthesis were enriched in species that host photosynthetic symbionts.

**Conclusions:**

Our results highlight the diverse functional roles that viruses can play in marine sponges and are consistent with our current understanding of sponge ecology. Differential representation of putative viral AMGs and accessory genes across sponge species illustrate the diverse suite of beneficial roles viruses can play in the functional ecology of these complex reef holobionts.

Video Abstract

## Introduction

Marine sponges (phylum Porifera) are an ecologically important component of the benthos, providing habitat for a diverse array of macro and microorganisms and mediating biogeochemical fluxes by filtering organic matter and facilitating the consumption and release of nutrients [1]. As suspension feeders, sponges can filter up to 100,000 times their own body volume in seawater every day [2], which influences the composition of the seawater at macro and micro scales [3–5]. Sponges efficiently extract picoplankton, bacteria and archaea [6], and can also retain viral-sized particles [7]. Moreover, most sponge species host diverse and stable communities of microbial symbionts, which contribute to a variety of host metabolic processes and produce a suite of secondary metabolites [8–11]. Although the complexity and composition of the microbiome varies across different sponge species, a recent survey of Indo-Pacific reef sponges revealed enrichment of several microbial phyla including the *Proteobacteria* (classes Alpha- and *Gammaproteobacteria*), *Actinobacteria*, *Chloroflexi*, *Nitrospirae* and *Cyanobacteria*, with *Thaumarchaeota* being the primary sponge-associated archaeal taxa [12]. Additionally, the microbiome of cosmopolitan sponges, such as *Carteriospongia foliascens* and *Xestospongia testudinaria*, often shows biogeographic distinctions, likely responding to environmental variations [13, 14]. Sponges and their complex communities of microbial symbionts are therefore a typical example of a ‘meta-organism’ or ‘holobiont’ [15, 16]. However, whilst sponge-microbial interactions have been extensively studied over the past decades [12, 17–19], viruses represent the ‘dark matter’ in these ecologically important symbioses.

Viruses are recognised as the most abundant entity in marine environments, likely infecting all organisms in the ocean [20, 21] and directly affecting energy flux in marine food webs via their regulation of prokaryotic and eukaryotic populations [22–24]. Despite the critical role of viruses in marine ecosystems, we are only just beginning to describe their diversity and contributions to host ecology. This is particularly important considering the recently recognised role of phages in manipulating their bacterial hosts due to alteration of host metabolism or host-microbial interactions via auxiliary metabolic genes (AMGs) or accessory genes. AMGs consist of a variety of host-derived genes with broad functional diversity that can contribute to the metabolism of their cellular hosts via processes including photosynthesis, nucleotide metabolism and nutrient cycling [25–27]. In addition to influencing host molecular processes during the viral infection cycle, it has been suggested that AMGs may play key roles in the environmental adaptation of their hosts [28].

Viral-like particles (VLPs) in sponges were first reported from transmission electron micrographs in 1978 [29]; however, it was not until 2016 that computational tools were optimised to explore sponge-associated viruses using viromic sequencing [30]. A subsequent comparative viromic analysis of coral and sponge-associated viruses revealed high intra-species similarity in the viromes of four sponge species, with communities dominated by double-stranded DNA (dsDNA) bacteriophage of the order *Caudovirales*, and a diverse community of single-stranded DNA (ssDNA) viruses of the family *Microviridae* [31]. Viruses belonging to the order *Megavirales* were also consistently observed, including members of the *Mimiviridae*, *Phycodnaviridae* and *Poxviridae* families [31]. A recent study, which assessed the RNA virome of the sponge *Hymeniacidon* sp. using dsRNA and ssRNA-seq also revealed a diverse RNA viral population, with matches to *Totiviridae*, *Reoviridae* and *Partitivirdae* [32]*.* Viromic studies have also provided important insights into how the viral community changes in diseased or stressed sponges [33–35], with thermal stress leading to an enrichment of endogenous retro-transcribing viruses in *Rhopaloeides odorabile* [35] and dysbiosis in the virome occurring in diseased *Lubomirskia baikalensis* [34]. Putative AMGs have also been detected in the viromes of some reef sponges. For instance, cobalamin biosynthesis and herbicide resistance genes were detected in the viromes of some Great Barrier Reef (GBR) sponge species [31] and an ankyrin domain-containing protein was discovered in symbionts of Mediterranean sponges, which upon bacterial expression dampened the eukaryotic immune response and altered host phagocytic activity, suggesting a role for the putative AMG in facilitating host-microbe interactions [36].

**Fig. 1:**
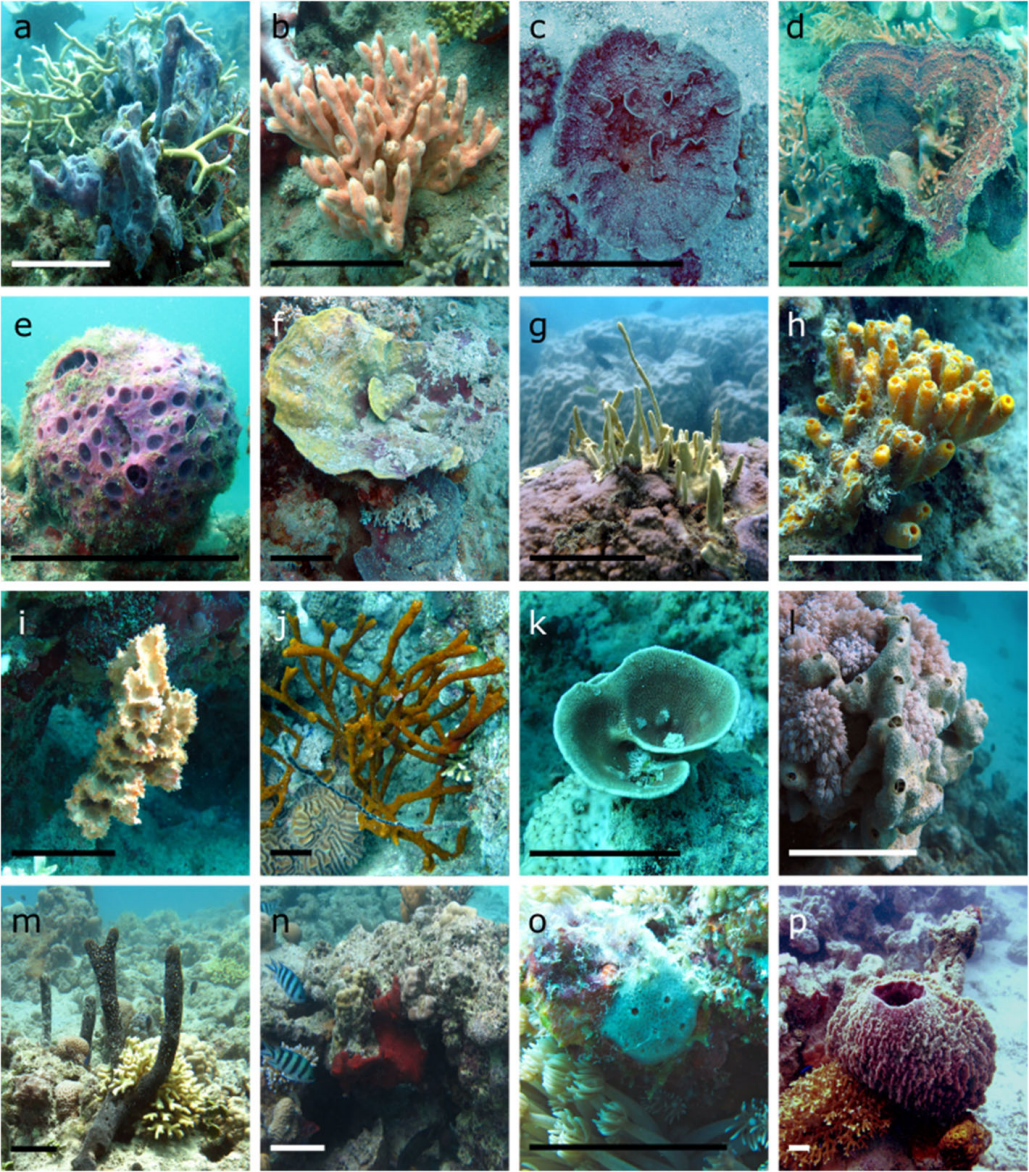
Sponge species used for viromic analysis. GBR sponges: *Callyspongia* sp. (**a**), *Echinochalina isaaci* (**b**), *Carteriospongia foliascens* (**c**), *Ianthella basta* (**d**), *Cinachyrella schulzei* (**e**), *Cymbastella marshae* (**f**), *Lamellodysidea herbacea* (**g**), *Pipestela candelabra* (**h**), *Sylissa carteri* (**i**); and the Red Sea sponges: *Amphimedon ochracea* (**j**), *Carteriospongia foliascens* (**k**), *Crella cyathophora* (**l**), *Hyrtios erectus* (**m**), *Mycale* sp. (**n**), *Niphates* sp. (**o**), *Xestospongia testudinaria* (**p**). Scale bar = 10 cm

To assess the ubiquity of putative AMGs and accessory genes in sponges and investigate how these viruses contribute to host ecology, we undertook deep viromic sequencing of 15 representative sponge species (Fig. 1) from two coral reef ecosystems, the GBR and the Red Sea.

## Results

### Community profile of the sponge virome

In total, 575,118 contigs were assembled and 1,162,879 genes were predicted (Table 1; Additional file 1). On average, 19.24% of all predicted genes were taxonomically assigned and 27.29% of all contigs contained at least one taxonomically assigned gene (Table 1; Additional file 1). Cellular marker evaluation identified that an average of 0.25% of contigs contained cellular marker matches (Additional file 1), comparable to a previous study which reported that host-associated viromes with 0.1-0.3% of contigs containing cellular marker matches could be characterised as having negligible or low-level cellular contamination [31]. Importantly, viromes presented here are based on homology comparisons to known viral genomes, an approach that cannot provide unequivocal taxonomic assignment of novel viruses.

**Table 1:** Summary of sampling locations, sponge features classified according to (i) host nutritional mode (presence or absence of photosymbionts); (ii) microbial symbiont strategy (high or low microbial abundance) and sequencing statistics. N50 values for each dataset were calculated based on evaluation of unfiltered contigs

Host species/replicate	Sampling site^1^	Hosts photo-symbionts	Microbial abundance	Raw reads* (#)	Contig N50	Contigs (#)	Longest contig (bp)	Predicted genes (#)	Taxonomically assigned genes (%)
*Callyspongia sp.* 1	*GBR*	Yes	LMA	11.06	899	23,339	58,159	63,281	23.5
*Callyspongia sp.* 2	*GBR*	Yes	LMA	9.36	932	20,309	172,832	56,308	23.6
*Callyspongia sp.* 3	*GBR*	Yes	LMA	8.97	810	21,236	69,654	56,363	24.0
*C. foliascens* 1	*GBR*	Yes	HMA	4.92	675	33,078	24,096	57,288	13.2
*C. foliascens* 2	*GBR*	Yes	HMA	1.78	698	14,225	46,996	25,768	11.0
*C. foliascens* 3	*GBR*	Yes	HMA	15.28	624	45,237	23,964	76,516	13.0
*C. schulzei* 1	*GBR*	No	HMA	7.95	467	9621	80,905	17,001	14.8
*C. schulzei* 2	*GBR*	No	HMA	8.23	571	12,170	81,025	26,002	17.5
*C. schulzei* 3	*GBR*	No	HMA	19.59	660	50,864	80,778	95,389	14.4
*C. marshae* 1	*GBR*	Yes	HMA	1.53	434	4549	9804	7975	16.2
*C. marshae* 2	*GBR*	Yes	HMA	3.75	398	5419	7498	6585	16.6
*C. marshae* 3	*GBR*	Yes	HMA	3.12	405	6751	12,455	10,427	16.1
*E. isaaci* 1	*GBR*	NA	NA	4.20	566	11,349	31,106	21,794	22.5
*E. isaaci* 2	*GBR*	NA	NA	4.50	510	13,898	49,604	28,567	23.3
*E. isaaci* 3	*GBR*	NA	NA	1.26	694	4752	42,237	11,022	21.8
*I. basta* 1	*GBR*	No	LMA	6.58	504	11,600	60,819	24,285	19.3
*I. basta* 2	*GBR*	No	LMA	4.63	495	12,591	39,149	23,850	22.2
*I. basta* 3	*GBR*	No	LMA	4.83	552	14,561	36,340	28,801	20.8
*L. herbacea* 1	*GBR*	Yes	LMA	7.96	457	11,693	43,955	21,907	20.2
*L. herbacea* 2	*GBR*	Yes	LMA	14.88	594	9661	14,898	18,144	19.2
*L. herbacea* 3	*GBR*	Yes	LMA	3.02	504	16,931	43,769	30,283	20.2
*P. candelabra* 1	*GBR*	NA	NA	12.20	693	35,688	49,806	32,910	15.0
*P. candelabra*2	*GBR*	NA	NA	7.74	557	27,010	58,681	48,732	14.4
*P. candelabra* 3	*GBR*	NA	NA	5.87	958	13,373	120,555	29,643	18.2
*S. carteri* 1	*GBR*	No	LMA	6.34	346	4063	10,851	5709	17.6
*S. carteri* 2	*GBR*	No	LMA	4.78	384	7833	16,548	13,143	21.9
*A. ochracea* 3	Red Sea	Yes	LMA	3.38	720	7706	26,892	18,040	25.6
*A. ochracea*1	Red Sea	Yes	LMA	14.14	717	29,033	61,876	71,543	23.2
*A. ochracea* 2	Red Sea	Yes	LMA	2.79	694	7692	20,756	10,981	21.1
*C. foliascens* 1	Red Sea	Yes	HMA	2.30	1671	2571	35,948	7096	20.7
*C. foliascens* 2	Red Sea	Yes	HMA	1.64	1135	3913	49,410	10,708	19.7
*C. foliascens* 3	Red Sea	Yes	HMA	1.82	1280	2787	38,097	7284	21.4
*C. cyathophora* 1	Red Sea	Yes	LMA	3.19	601	2757	19,321	8636	25.6
*C. cyathophora* 2	Red Sea	Yes	LMA	3.58	618	2613	39,999	5753	24.9
*C. cyathophora* 3	Red Sea	Yes	LMA	1.84	614	3977	44,834	7951	21.5
*H. erectus* 1	Red Sea	No	HMA	18.37	921	6400	7865	17,674	21.8
*H. erectus* 2	Red Sea	No	HMA	6.04	878	9540	47,148	25,550	23.9
*Mycale sp.* 1	Red Sea	NA	NA	5.81	949	5396	39,546	13,458	16.1
*Mycale sp.*2	Red Sea	NA	NA	5.74	705	9562	54,476	22,104	22.7
*Mycale sp.* 3	Red Sea	NA	NA	5.06	682	6123	22,927	13,601	20.2
*N. rowi* 1	Red Sea	NA	NA	2.40	668	6058	25,175	13,766	27.5
*N. rowi* 2	Red Sea	NA	NA	7.35	922	10,017	52,589	27,503	24.0
*X. testudinaria* 1	Red Sea	Yes	HMA	6.13	881	7533	103,486	20,344	16.2
*X. testudinaria* 2	Red Sea	Yes	HMA	7.40	729	9639	74,732	23,194	17.1
*Sea water—GBR*	GBR	NA	NA	21.64	406	11,008	20,625	19,950	22.0
*Sea water—RS*	Red Sea	NA	NA	9.51	523	10,568	52,589	36,395	35.1

^1^GBR sponges were collected from Orpheus Island, Queensland, Australia (18° 35′ 34″ S, 146° 28′ 53″ E) and Red Sea sponges were collected in Al Fahal, Saudi Arabia (22° 13′ 95″ N, 39° 01′ 81″ E)

*Raw read values are presented as million of reads. Taxonomic assignments are based on pairwise sequence similarity to the Viral RefSeq database using MEGAN LCA parameters [30]

**Fig. 2:**
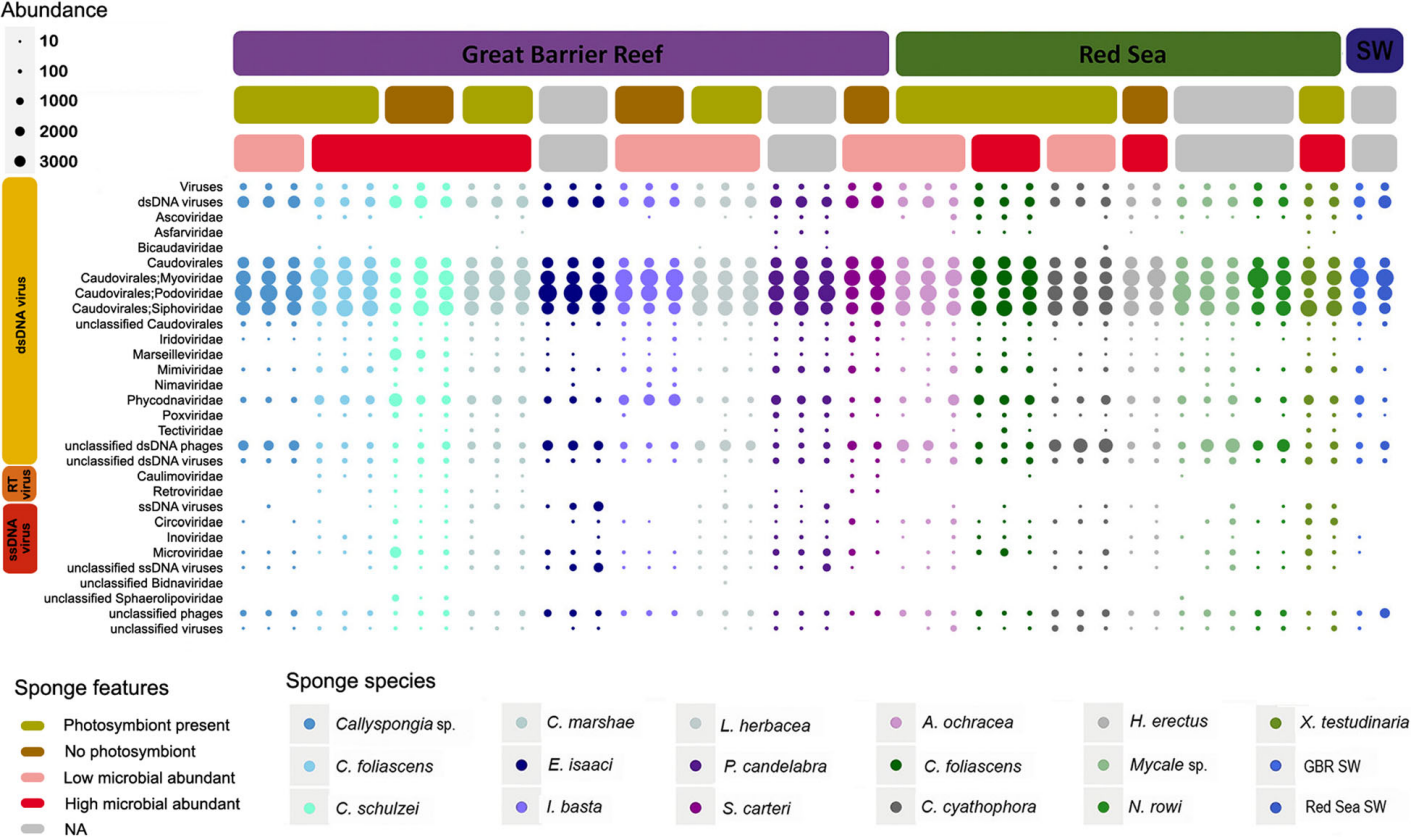
Taxonomic summary of the viral communities associated with coral reef sponges. Column headings display nine sponge species from the Great Barrier Reef and seven from the Red Sea. The top 30 most frequent taxonomic assignments are summarised at the family level based on a normalised comparison (normalised gene count ~ 33,250 per dataset) of viral RefSeq gene assignments in MEGAN6 using parameters defined in Laffy et al. [30]. Abundance for each viral taxa was calculated using contig coverage estimates to identify proportional representation within each dataset. Only viral sequences that underwent taxonomic assignment and datapoints with abundance values of 10 or more were included within this plot. RNA viral taxonomic assignments were filtered from the final dataset

All abundance values for assembled contigs were adjusted as described in the HoloVir workflow [30], where read coverage values were considered by MEGAN in the calculation of relative abundance values for each contig. A detailed evaluation of the HoloVir gene-centric annotation process identified that assembled viromes contain fewer cellular contaminants and more accurately represent viral assemblages [31]. It is worth noting that both taxonomic and functional analysis of the viromes is based on the proportion of total genes that could undergo taxonomic or functional assignment, hence, does not reflect absolute community composition. Furthermore, all taxonomic assignments were performed using the viral component of the RefSeq database, which is unlikely to identify cellular gene matches. Sponge-derived viral sequences predominantly matched dsDNA viruses (88%), with a lower relative abundance of matches to ssDNA viruses (9%) and retroviruses (3%) (Fig. 2; Additional file 2). In particular, matches to the tailed bacteriophage order *Caudovirales*, including representatives of the *Podoviridae*, *Siphoviridae*, and *Myoviridae*, accounted for more than 80% of total viral taxonomic assignments (Fig. 2; Additional file 2). Contigs taxonomically assigned to viral families that typically infect eukaryotes were also prevalent in sponges, particularly representatives of the nucleocytoplasmic large DNA virus (NCLDV) families *Mimiviridae*, *Marseilleviridae*, *Phycodnaviridae*, *Ascoviridae*, *Iridoviridae*, *Asfarviridae* and *Poxviridae*. Matches to the ssDNA viral families *Microviridae*, *Circoviridae* and *Inoviridae* were evident in most sponge species whereas the *Bidnaviridae* had a more restricted distribution and lower intra-species similarity than other viral taxa (Fig. 2). Retroviral sequences assigned to the families *Caulimoviridae* and *Retrovirdae* were also detected in just over one-third of sponge species, including all replicates of the GBR sponges *Carteriospongia foliascens*, *Cinachyrella schulzei*, *Cymbastela marshae* and *Stylissa carteri* (Fig. 2).

### Variation in the sponge viral community is driven by site-specific and host-specific features

**Fig. 3:**
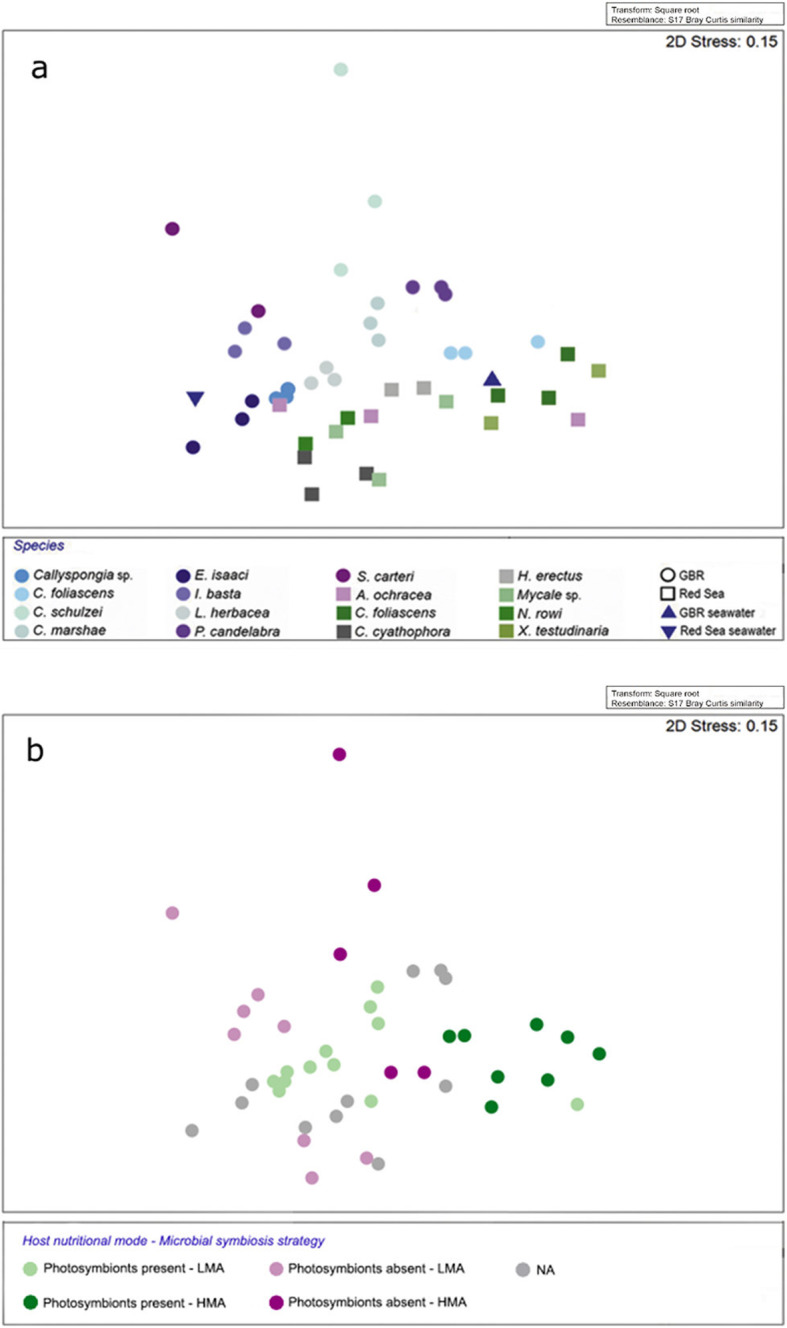
Endogenous and exogenous determinants of taxonomically assigned viral community composition within marine sponges. Non-metric multidimensional scaling plot based on Bray-Curtis similarity of genus-level taxonomy for predicted genes. Ordination displays similarities in the viral communities of the fifteen sponge species (PERMANOVA, pseudo-*F* value = 3.9437, df = 14, *P* value ≤ 0.001) from the Great Barrier Reef and the Red Sea (PERMANOVA, pseudo-*F* value = 11.354, df = 1, *P* value ≤ 0.001) (**a**), and discriminates between species classified as high microbial abundance (HMA) or low microbial abundance (LMA) (PERMANOVA, pseudo-*F* value = 6.0159, df = 1, *P* value ≤ 0.001) and host nutritional modes, classified by the presence or absence of photosymbionts (PERMANOVA, pseudo-*F* value = 3.2176, df = 1, *P* value = 0.007) (**b**)

The composition of sponge-associated viral communities varied by host species and host geographic location (Fig. 3a). A significant difference in viral community composition was found between the 15 sponge species (PERMANOVA, pseudo-*F* value = 3.9437, df = 14, *P* value ≤ 0.001, Fig. 3a), as well as between the two sampling sites (PERMANOVA, pseudo-*F* value = 11.354, df = 1, *P* value ≤ 0.001; Fig. 3a). Pairwise PERMANOVA comparisons also revealed specific differences in the viral communities across sponge species within each respective location (Additional file 3: Table S1). For instance, in the GBR, *C. foliascens*, *Echinochalina isaaci* and *Ianthella basta* had viral community profiles that were significantly different to all other sponge species (Additional file 3: Table S1). *Callyspongia* sp. had the most conserved viral community amongst biological replicates, with over 91% community similarity across samples (Additional file 3: Table S2). In the Red Sea, significant differences in viral populations were detected between *C. foliascens* and the sponges *Crella cyathophora*, *Hyrtios herectus*, *Mycale* sp. and *Niphates rowi*, as well as between *C. cyathophora* and *Xestospongia testudinaria* (Additional file 3: Table S1). In the Red Sea, *C. foliascens* had the highest intra-species similarity, with biological replicates sharing 86% similarity in their viral communities (Additional file 3: Table S3). Viral communities were also more similar amongst sponges sharing similar traits in microbial ecology (Fig. 3b; Additional file 4), with permutation-based analysis of variance revealing significant differences in viral community composition between high microbial abundance (HMA) and low microbial abundance (LMA) sponge species (PERMANOVA, pseudo-*F* value = 6.0159, df = 1, *P* value ≤ 0.001) and between sponges with and without photosymbionts (PERMANOVA, pseudo-*F* value = 3.2176, df = 1, *P* value = 0.007). SIMPER analysis further revealed that viromes of all HMA species shared 70% similarity whereas the viromes of LMA species shared 72%, with higher abundances of bacteriophage taxa in HMA species contributing to 43% of the total dissimilarity between these groups (Additional file 4). Sponges with photosymbionts shared 71% similarity in their viromes whereas sponges without photosymbionts shared 67%, with higher relative abundances of algal viruses contributing to the dissimilarity between host nutritional modes (Additional file 4).

### Visualisation of sponge-associated viral-like particles (VLPs) by TEM

**Fig. 4:**
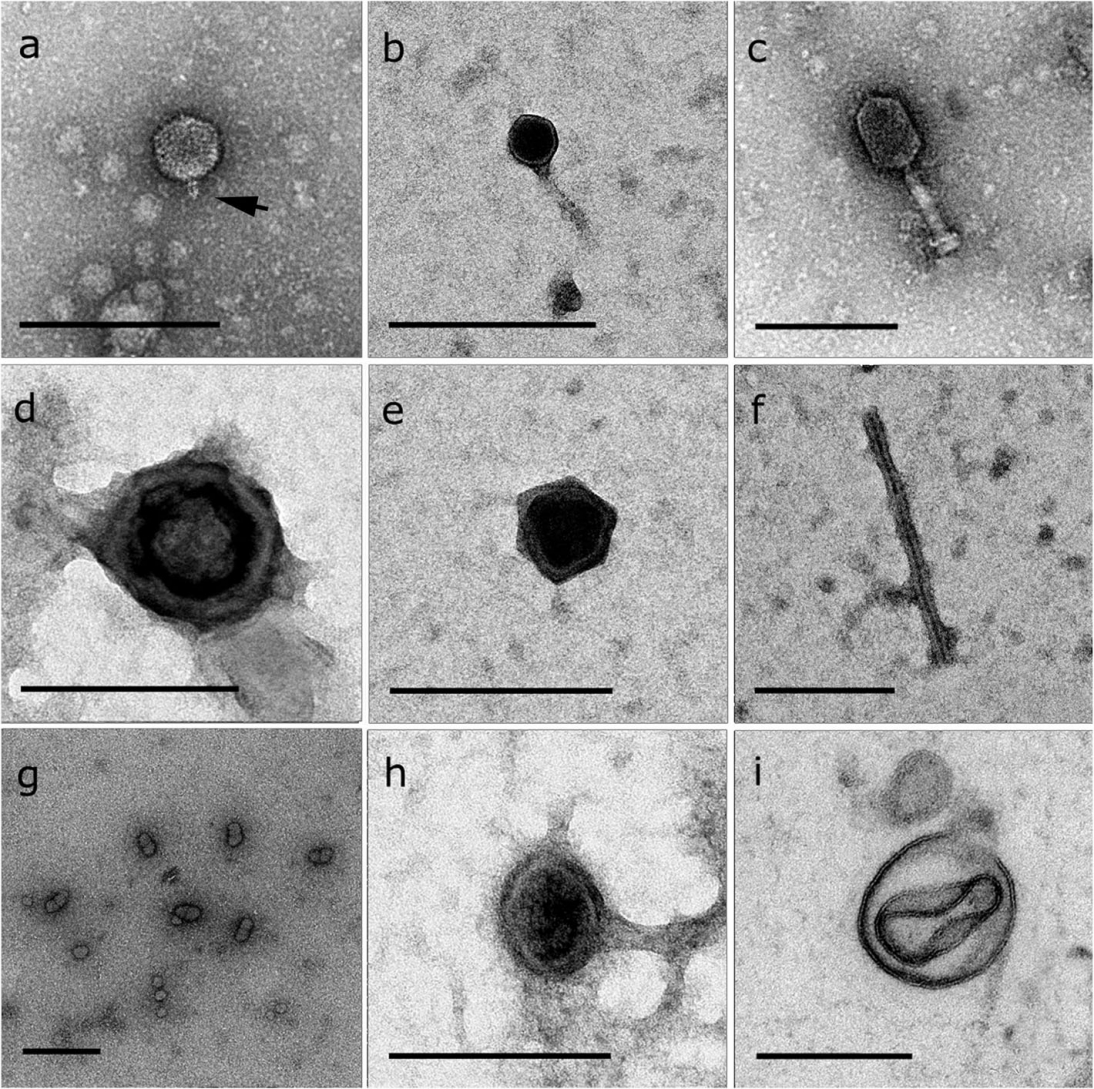
Transmission electron micrographs depicting representative viral like particles (VLPs) associated with coral reef sponges. Representative tailed VLPs in *Stylissa carteri* (**a**, **c**) and *Amphimedon ochracea* (**b**); non-tailed icosahedral VLPs in *Pipestela candelabra* (**d**) and *Cinachyrella schulzei* (**e**); filamentous VLP from *Cinachyrella schulzei* (**f**); Geminate VLPs from *Amphimedon ochracea* (**g**); lemon-shaped VLP from *Carteriospongia foliascens* (**h**) and brick-shaped VLP from *Crella cyathophora* (**i**); using the TEM preparation methods: sponge sections (**i**) CsCl gradient separation (**d**, **g**) and from surface mucus (**a**-**c**, **e**-**f**, **h**). Scale bar = 100 nm. Arrow in Fig. 4a denotes the presence of a capsid tail structure

Transmission Electron Microscopy (TEM) was used to visualise viral groups detected within samples investigated via viromic sequencing. TEM revealed a broad range of viral morphologies spanning tailed VLPs (Fig. 4a-c), non-tailed icosahedral VLPs (Fig. 4d, e), filamentous VLPs (Fig. 4f), putative geminated VLPs (Fig. 4g), putative lemon-shaped VLPs (Fig. 4h) and brick-shaped VLPs (Fig. 4i). Viral-like particles were observed within sponge cells, mesohyl, mucus/surface biofilm and within the sponge-associated microorganisms. Most VLP morphotypes showed icosahedral/polyhedral capsid symmetry. Amongst the VLPs with icosahedral capsids, tailed viruses were classified based on tail size/shape as belonging to the three *Caudovirales* families: *Podoviridae*, *Siphoviridae* and *Myoviridae* [37] (Fig. 4a-c). *Podoviridae* VLPs presented a short tail attached to a non-enveloped icosahedral capsid (Fig. 4c). *Siphoviridae* VLPs showed a symmetric icosahedral capsid with a clearly distinct electro-dense core and long tail (Fig. 4b). Lastly, *Myoviridae* VLPs resembled T4-like bacteriophage with elongated hexagonal capsid head and tail connected with the head through a neck and a basal plate on the opposite tail extremity (Fig. 4c). Brick-shaped VLPs detected in the sponge mesohyl were morphologically consistent with members of the *Poxviridae* family (Fig. 4i) [38].

### Functional potential of the sponge virome

On average, 14.6% of predicted genes from the sponge viromes were assigned functional Swiss-Prot keywords, based on BLASTP matches to the UniProt-KB database (Additional file 1). Ordination analysis based on the relative frequency of Swiss-Prot keywords revealed both species-specific and site-specific clustering in genes coding a related function (Additional file 3: Fig. S1) (Fig. 3a). Permutational analysis of variance confirmed significant differences in functional gene repertoires across species (PERMANOVA, pseudo-*F* value = 4.4106, *P* value ≤ 0.001) and locations (PERMANOVA, pseudo-*F* value = 11.271, *P* value ≤ 0.001). Pairwise PERMANOVA comparisons of Swiss-Prot keyword abundance data across GBR and Red Sea sponge viromes showed that overall, Red Sea sponges had lower variation in the relative frequency of Swiss-Prot keywords across species than GBR sponges (Additional file 3: Table S4). Viral functional profiles of all GBR sponges were distinct between species, although only *C. foliascens* and *I. basta* were significantly different from every other sponge species (Additional file 3: Table S4). A full list of intra and inter-species functional similarity can be found in Additional file 3: Tables S5-S6.

**Fig. 5:**
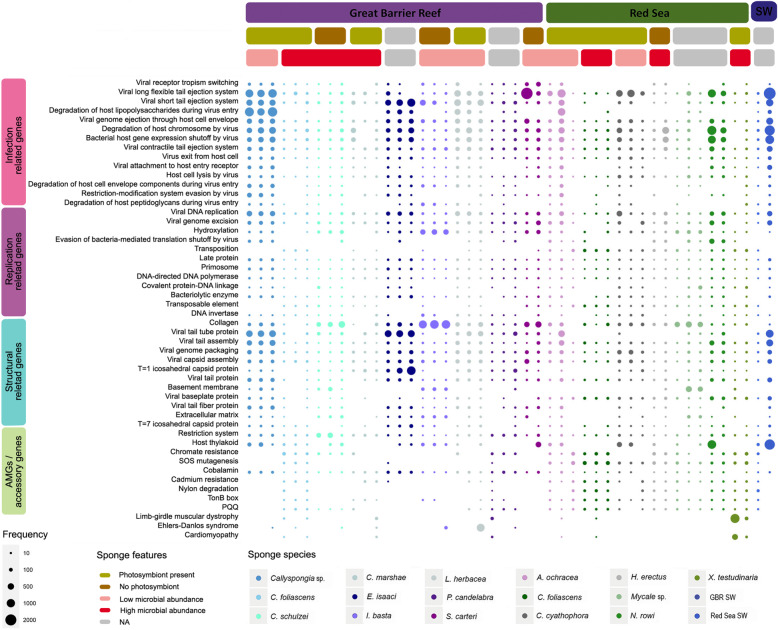
The top 50 most abundant keywords across all virome datasets associated with coral reef sponges. Swiss-Prot keyword frequency was calculated for each virome by adjusting for contig coverage combined with the overall frequency of each keyword within the Swiss-Prot database and an *e* value cutoff of 1e^−10^. Only keywords with a frequency value greater than 2 are displayed within each dataset and keywords are presented sorted by viral functional gene categories, including viral infection, replication, structural formation and putative AMG manual classifications, further sorted by overall frequency values within each category

Each sponge species showed a unique functional profile (Fig. 5); however, of the 50 most enriched Swiss-Prot keywords, half were abundant across all sponge samples, whilst the remaining keywords were enriched only in specific sponge species (Fig. 5). Of the 50 most enriched viral keywords, 28% were associated with viral infection strategies including ‘genome ejection through the host cell envelope’, ‘attachment to host entry receptor’, ‘long flexible tail ejection system’ and ‘exiting from the host cell’ (Fig. 5). A further 24% were involved in viral structure, including ‘*t* = 1 icosahedral capsid protein’, ‘collagen’, ‘tail assembly’ and ‘tail protein’ (Fig. 5). Additionally, viral replication mechanisms comprised 24% of the top 50 keywords, including ‘DNA replication’, ‘genome excision’, ‘DNA invertase’ and ‘DNA-directed DNA polymerase’ (Fig. 5). Finally, 18% of the 50 most enriched protein functions related to a suite of putative AMGs and accessory genes, including ‘chromate resistance’, ‘cadmium resistance’, ‘nylon degradation’, ‘SOS mutagenesis’ and ‘host thylakoid’ (Fig. 5).

**Fig. 6:**
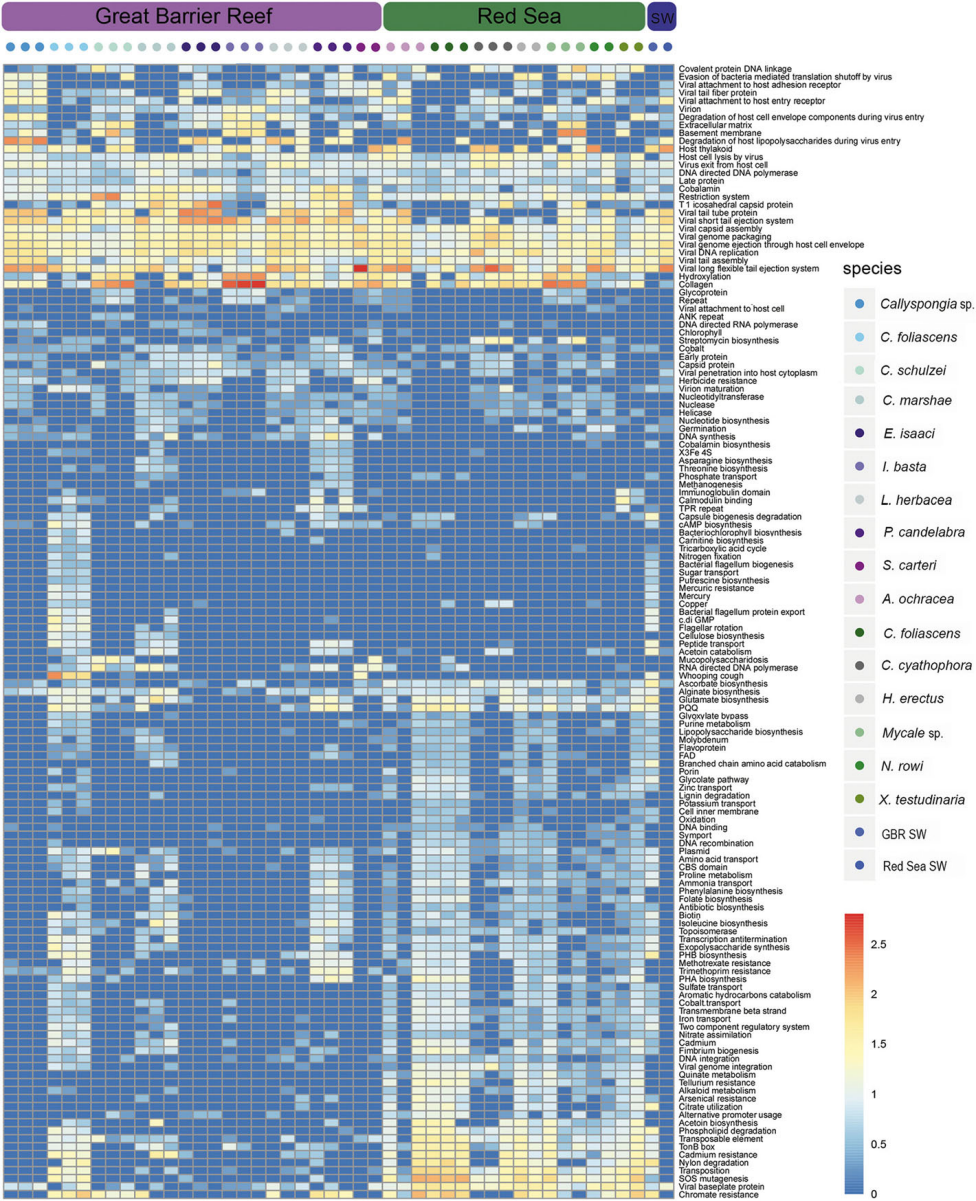
Drivers of viral functional variation between sponge species. To identify key functional differences in viromes of each sponge species, the R package mvabund was used to perform univariate tests on Swiss-Prot keyword enrichment frequency values. Heatmap shows all significant differences in Swiss-Prot keyword enrichment frequency data (*P* value ≤ 0.02), adjusted to account for coverage of the source contig within individual viromes

**Fig. 7:**
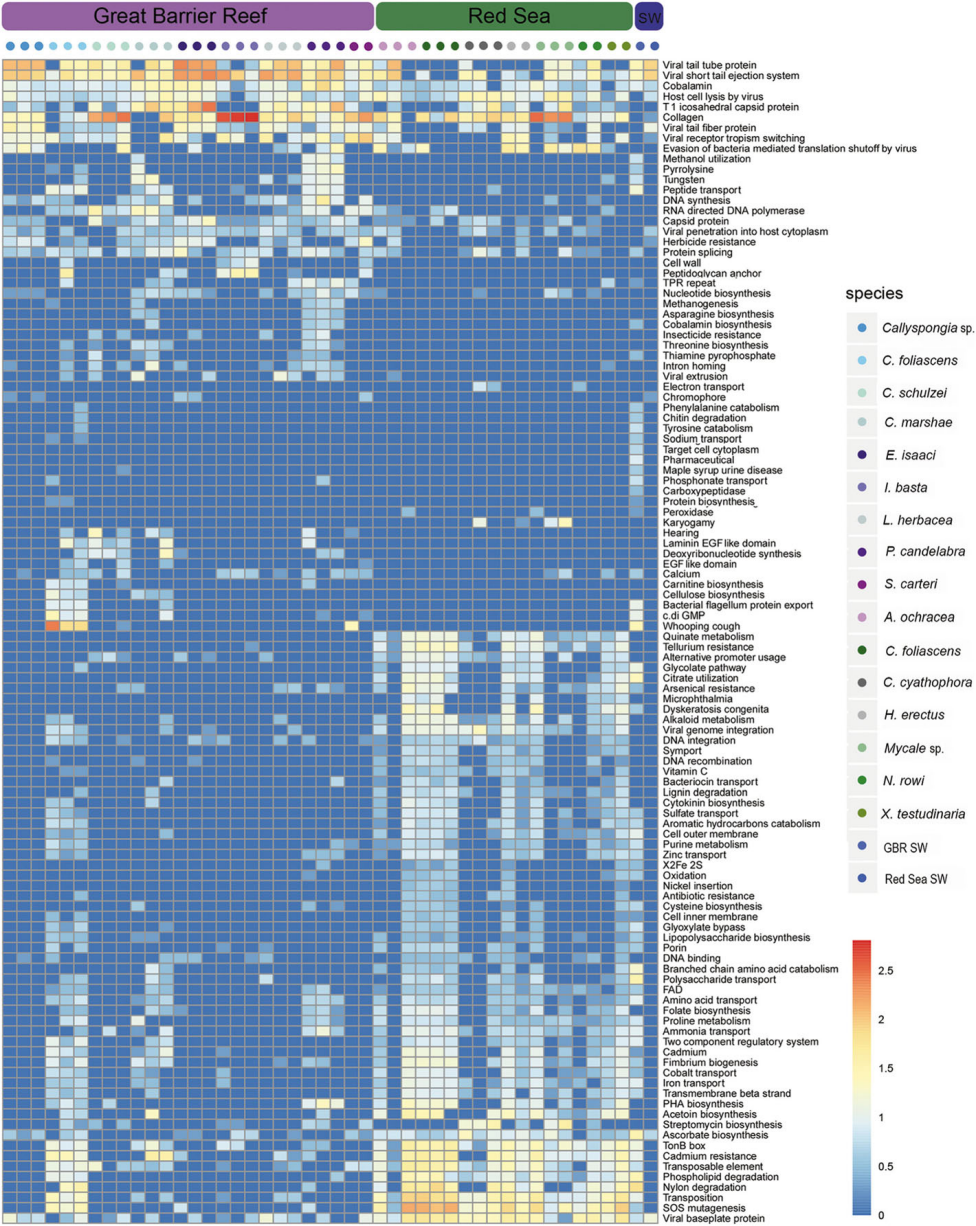
Drivers of viral functional variation between sampling sites. To identify key functional differences in sponge viromes between the Great Barrier Reef and Red Sea, the R package mvabund was used to perform univariate tests on Swiss-Prot keyword enrichment frequency values. Heatmap shows all significant differences in Swiss-Prot keyword enrichment frequency data (*P* value ≤ 0.02), adjusted to account for coverage of the source contig within individual viromes

Significant differences in specific viral functions between host species (Fig. 6) and sampling sites (Fig. 7) were identified using mvabund analysis of the Swiss-Prot functional keywords. The Swiss-Prot keyword ‘host thylakoid’ was particularly enriched in *Callyspongia* sp., *C. foliascens*, *C. schulzei*, *I. basta*, *S. carteri*, *C. cyathophora* and *N. rowi* (Fig. 6). Genes associated with this keyword encode a Photosystem II D2 protein, and the majority of contigs with this gene originated from *Synechococcus* phages within the family *Myoviridae* (Additional file 5). Although these genes are known to be associated with photosynthetic responses, not all sponge species hosting photosymbionts were enriched in viral-derived photosystem II proteins (e.g. *C. marshae*, *L. herbacea*, *A. ochracea*; summarised in Additional file 5), consistent with previous research showing that not all cyanophages carry these genes [39]. Predicted collagen proteins were enriched in all sponge viromes (Fig. 5) and were a significant driver of functional differences between host species (Fig. 6) and sampling locations (Fig. 7). Contigs encoding predicted collagen proteins were consistently attributed to dsDNA viruses (Additional file 5), and when assigned at the family level, included members of the bacteriophage families *Myoviridae*, *Podoviridae* and *Siphoviridae*, the algal *Phycodnaviridae*, the crustacean-infecting *Nimaviridae* and the giant virus family *Mimiviridae* (Additional file 5). Genes coding for ankyrin repeat proteins (ARPs) were found on 60 contigs within the *C. schulzei* viromes, and 65% of these were taxonomically assigned to contigs matching dsDNA viruses (Additional file 5).

Heavy metal resistance genes, including mercury, molybdenum, chromate, cadmium, tellurium and arsenic resistance were significantly enriched in *C. foliascens*, *X. testudinaria*, *C. schulzei*, *H. erectus* and *P. candelabra* and more broadly in sponges from the Red Sea sampling site (Figs. 6 and 7). Genes associated with arsenic resistance (arsenite and arsenate reductase genes) were significantly enriched in Red Sea sponges (Figs. 6 and 7), with the source contigs being almost exclusively assigned to bacteriophage (Additional file 5). Tellurium resistance was also detected in all Red Sea sponges and was a significant driver of functional differences between the viromes at each location (Fig. 7). Contigs carrying tellurium resistance genes were primarily assigned to the bacteriophage family *Myoviridae* (Additional file 5). With the exception of *C. cyathophora*, chromate resistance genes were significantly enriched in all Red Sea sponges, as well as in five of the nine GBR sponges. The chromate resistance keyword was assigned to multiple genes included in operons containing both chromate and molybdite resistance [40, 41] on contigs taxonomically assigned as *Caudovirales* (Additional file 5). Similarly, cadmium resistance genes were significantly enriched in Red Sea sponges (Fig. 7). For the few contigs containing cadmium resistance genes that could be taxonomically assigned, matches were made to dsDNA viruses from the *Caudovirales* and *Phycodnaviridae* (Additional file 5). An enrichment of Swiss-Prot functional keywords for nylon degradation was also detected in the Red Sea sponge viromes with contigs taxonomically assigned to *Siphoviridae* (Additional file 5). In contrast to the putative AMGs enriched in the Red Sea environment, herbicide resistance genes were significantly enriched in the GBR (Fig. 7). Genes related to herbicide resistance were primarily assigned to contigs from *Synechococcus* phages and VirSorter analysis supported their viral origin (Additional file 5).

Analysis of how virome function reflected other aspects of host ecology revealed significant differences according to host nutritional mode (photosymbionts vs no photosymbionts; PERMANOVA, pseudo-*F* value = 2.1976, df = 1, *P* value ≤ 0.001) and microbial abundance (HMA vs LMA; PERMANOVA, pseudo-*F* value = 2.4712, df = 1, *P* value ≤ 0.001). Specific differences in viral functions were assessed by mvabund analysis of the Swiss-Prot keywords (Additional file 3: Figs. S2 and S3). The keyword ‘antimicrobial’ was significantly enriched in LMA sponges (Additional file 3: Fig. S2). Within the LMA sponge viromes containing antimicrobial genes, source contigs were assigned to members of the *Caudovirales* (Additional file 5), and most were linked to hydrolytic enzymes (Additional file 5). Additionally, significant enrichment of the Swiss-Prot keywords ‘nitrate assimilation’ and ‘nitrogen fixation’ were detected in HMA species (Additional file 3: Fig S2).

Significant differences in viral functional genes were also evident between the phototrophic and heterotrophic sponge species. For instance, the ‘cellulose biosynthesis’ keyword was significatively enriched in sponges hosting photosynthetic symbionts (Additional file 3: Fig. S3), particularly in *C. foliascens* and *C. marshae* (Additional file 5). Associated with the cellulose biosynthesis keyword were genes related to cellulose synthase A (CesA) and probable diguanylate cyclase genes, which were both assigned to *Phycodnaviridae* and *Myoviridae* (Additional file 5).

Critical evaluation of the origin of all putative AMGs and accessory genes was performed by cross-referencing contig level HoloVir taxonomic assignments, reporting source contig length and undertaking additional validation with VirSorter, a tool designed to identify viral contigs within cellular metagenomes [42]. VirSorter analysis confirmed that contigs containing genes with Swiss-Prot keyword assignments to antimicrobial activity, host thylakoid, herbicide resistance and collagen proteins were viral in origin (Additional file 5). VirSorter analysis did not provide additional support for contigs containing heavy metal resistance genes, nylon degradation, cellulose biosynthesis or nitrate fixation/assimilation, although it should be noted that VirSorter is typically unable to assess contigs less than 3 kb in length.

## Discussion

### Community profile of the sponge virome

Bacteriophage played a central role in structuring the sponge virome, since they were the dominant component of the viral communities. The *Caudovirales* infect a wide range of bacteria and archaea [43], are the most abundant viruses in marine environments [44], and have been reported to dominate the virome of numerous other coral reef species [31, 45–49]. The predominance of bacteriophage matches within the sponge viromes reflects the enormous abundance of microorganisms residing within the sponge holobiont, with as many as 10^9^ symbiont cells per cm^3^ of sponge tissue [50–52]. Although bacteriophage groups dominated the sponge viromes, contigs taxonomically assigned to members of viral families that typically infect eukaryotes were also prevalent, including representatives of the nucleocytoplasmic large DNA viruses (NCLDV). However, the presence and relative abundance of NCLDV assignments varied across sponge species (Fig. 2). *Mimiviridae* and *Marseilleviridae* are giant viruses that typically infect amoebae [53]. Whilst the sponge amoeba-like cells (amoebocytes and archaeocytes [54]) may host these NCLDV, giant viruses also associate with marine cnidarians, echinoderms and protochordates that lack typical amoebocyte cells [31, 46, 55–58]. The high relative abundance of Mimiviruses in marine waters [59] combined with their large genome sizes (~ 1.2 Mbp) may explain their prevalence in the sponge viromes. Conversely, sponge-derived Mimivirus-like contigs have low diversity and high species specificity [31], suggesting that the giant virus signature in sponges does not originate from seawater.

Matches to *Phycodnaviridae* were consistently detected across all fifteen sponge species (Fig. 2). Members of this viral family typically infect algae [60] and have been reported from cnidarian, arthropod, echinoderm and urochordate holobionts [45, 46, 61]. In sponges, the *Phycodnaviridae* are likely targeting the associated photosymbionts, which can occur at high abundance in many of these sponge species [62]. Another NCLDV family detected in the viromes of all sponge species was the *Poxviridae* (Fig. 2). *Poxviridae* and the viral families *Ascoviridae*, *Iridoviridae* and *Asfarviridae* are associated with a wide range of invertebrate hosts [57, 63–66]. The detection of *Poxviridae*-like viruses in marine sponges suggests an extension of their previously known host range, although cellular infection in sponges still needs to be validated. The NCLDV group of viruses pose considerable systematic and interpretative challenges due to horizontal gene transfer between different NCLDVs and their hosts, which can make taxonomic assignment hard to resolve [67].

The most prevalent ssDNA viral sequence assignments within the sponge viromes were to the *Microviridae*, which typically infect *Proteobacteria*, *Spiroplasma* and *Chlamydia* [43, 68]. Proteobacteria are abundant and diverse symbionts of marine sponges [69], likely explaining the high relative abundance and diversity of sequences assigned to these small ssDNA viruses in the sponge viromes as well as in viral communities from other reef invertebrates [31, 70]. The *Circoviridae* typically infect mammals and birds [71] but viruses from this family were also frequently detected in sponges. This group of viruses is characterised by their small circular genomes (~ 2 kb) and high genetic diversity, which has underpinned a rapid expansion in their host range [71, 72] to include cnidarians, urochordates and other invertebrates [31, 73].

Retroviral sequences assigned to the families *Caulimoviridae* and *Retrovirdae* were also detected in just over one-third of sponge species, including all replicates of the GBR sponges *C. foliascens*, *C. schulzei*, *C. marshae* and *S. carteri* (Fig. 2). Reverse-transcribing viruses infect a wide range of animal, algal and plant hosts [74–76] and have recently been reported within *Symbiodiniaceae* cultures from coral [45, 70]. The detection of retroviruses is not uncommon in viromic studies targeting DNA viruses [31, 46, 48, 77], and is possible because transcribed retroviral DNA can be present within retrovirus capsids, and this DNA can make up to 2.5% of the total virus nucleic acid [78]. Whilst our methodological approach should have precluded detection of RNA viruses, matches to the *Astroviridae* and *Coronaviridae* families were observed in several samples (Additional file 2), highlighting current methodological limitations in virome annotation. Lack of suitable genomic resources for accurate homology-based identification was likely also responsible for RNA viral annotations previously reported from DNA viromes recovered from corals [48]. However, it should be noted that assignment to RNA viruses made up a small proportion (< 0.5%) of all sponge samples with the exception of a single *C. foliascens* virome, where assignment to the *Coronaviridae* made up 1.8% of the total assigned viral community. The *C. foliascens* gene assigned as *Coronaviridae* originated from a contig with two other taxonomic assignments, both sharing homology with ssDNA *Microviridae* (data not shown). Further, VirSorter analysis identified this contig as viral in origin, with category 1 assignment (Additional file 5).

### Variation in the sponge viral community is driven by site-specific and host-specific features

The composition of sponge-associated viral communities was determined by host species and the geographic location of the host (Fig. 3a). The significant difference in viral community composition between the 15 sponge species is consistent with previous reports of high intra-species similarity in the viral communities of the sponges *Amphimedon queenslandica*, *Rhopaloeides odorabile*, *Xestospongia testudinaria* and *Ianthella basta* [31]. Given the large volumes of seawater sponges filter to extract bacterioplankton and virioplankton, this species specificity is particularly notable, and is likely attributed to the host specificity of eukaryotic viruses [57, 79–83] and the high species specificity of the sponge-associated microorganisms [12] that host the bacteriophage component of the community. The viral communities were also significantly different between sampling sites; however, this was unlikely attributed to differences between seawater viromes from the GBR and the Red Sea, since both coral reefs belong to the same ecological zone established by Gregory and colleagues [84], and are therefore expected to harbour similar viral taxa. The geographic variation in sponge-associated viruses is consistent with findings by Brum and colleagues [85], who reported that marine viral communities can be locally structured by specific environmental conditions that affect host community structure.

Permutation-based analysis of variance demonstrated that viral communities were more similar amongst sponges sharing similar traits in microbial ecology, revealing significant differences in viral community composition between HMA and LMA sponges and between sponges with and without photosymbionts. For instance, the Red Sea sponges that shared the highest similarity in their associated viral communities*, C. foliascens* and *X. testudinaria*, are both HMA species and associate with photosymbionts (Additional file 3: Table S3). Microbial biomass in HMA species can comprise up to one-third of the total sponge biomass, with microbial diversity generally being much higher than in sympatric LMA species [86–88]. A greater abundance of bacteriophage matches in HMA sponges was a major driver of the dissimilarity between HMA and LMA species (Additional file 4), further supporting the role of the sponge microbiome (abundance and composition) in structuring the virome.

### Functional potential of the sponge virome

Consistent with taxonomic analyses, ordination based on the relative frequency of Swiss-Prot keywords revealed both species-specific and site-specific clustering in gene function (Additional file 3: Fig. S1) where each sponge species showed a unique functional profile (Fig. 5). Marked host specificity in functional genes reflected the distinct viral communities inhabiting each of the holobionts. For instance, genes assigned the keyword for short tail ejection systems were particularly enriched in *E. isaaci* consistent with this species hosting the highest relative abundance of the short tail bacteriophage family *Podoviridae* (Fig. 2).

**Fig. 8:**
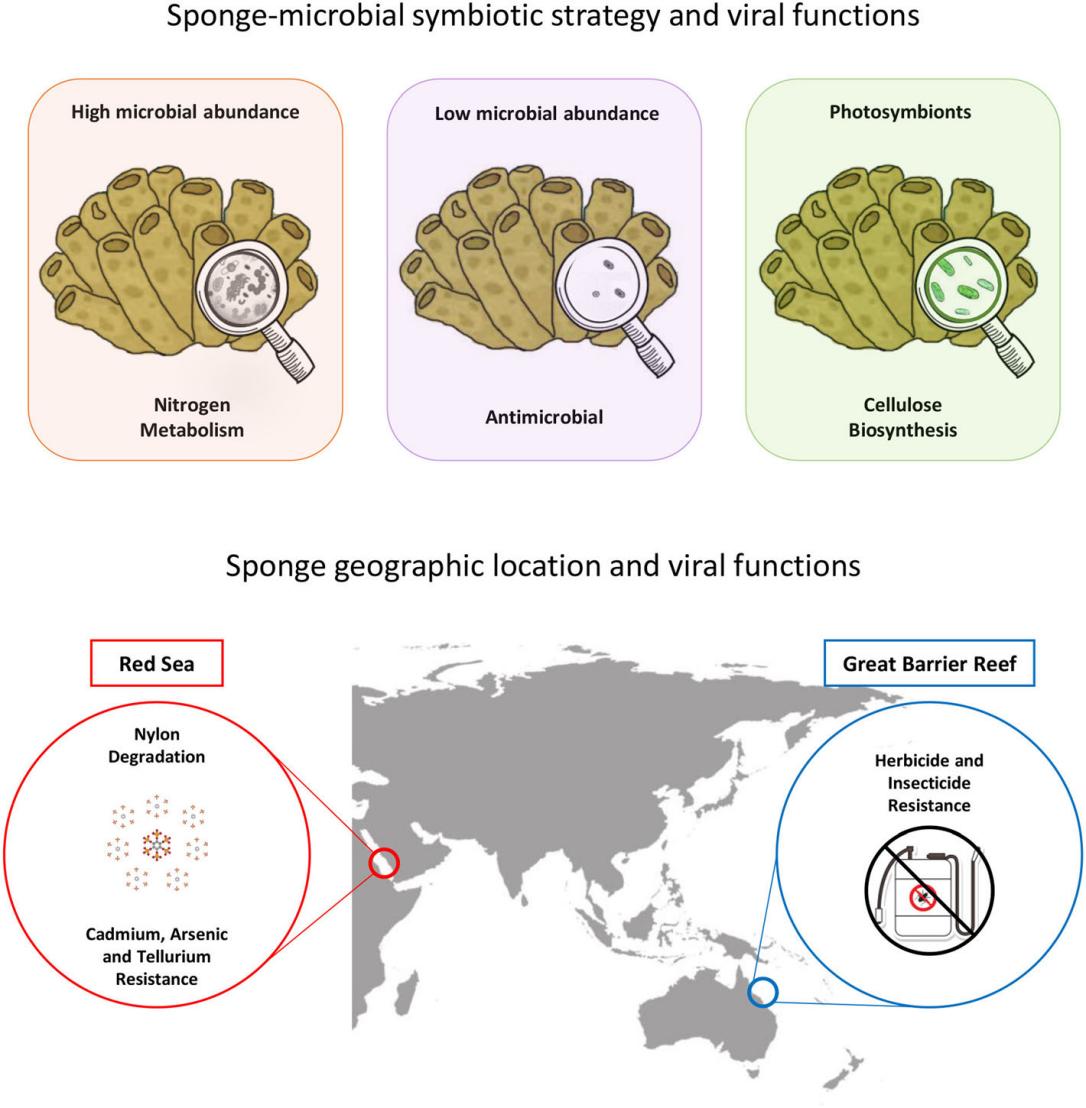
Comparison of viral function with sponge nutritional strategy, microbial abundance and geographic location. Comparison of viral functional profiles across 15 coral reef sponge species revealed that viral functions correlate with host nutritional strategy (photosymbionts vs no photosymbionts), microbial abundance (high microbial abundance—HMA, vs low microbial abundance—LMA) and geographic location (Fig. 3). Differential representation of putative AMGs and accessory genes across these ecological traits likely match host ecology. For instance, cellulose biosynthesis genes were enriched in phototrophic sponges, nitrogen metabolism genes were enriched in HMA sponges whilst antibiotic synthesis genes were enriched in LMA sponges. Additionally, heavy metal resistance genes were enriched in Red Sea sponges, whilst herbicide/insecticide resistance genes were enriched in GBR sponge viromes

Variation in putative AMGs and accessory genes between sponge species and sampling locations (Fig. 8) was supported by mvabund analysis, which identified significant differences in specific viral functions between host species (Fig. 6) and sampling sites (Fig. 7). For instance, genes assigned the ‘host thylakoid’ Swiss-Prot keyword, which is attributed to a protein located in or on the host thylakoid of chloroplasts of green algae [89], were particularly enriched in *Callyspongia* sp., *C. foliascens*, *C. schulzei*, *I. basta*, *S. carteri*, *C. cyathophora* and *N. rowi* (Fig. 6). The ‘host thylakoid’ genes shared sequence homology to known Photosystem II D2 proteins, and the majority of contigs containing these genes originated from *Synechococcus* phages within the family *Myoviridae* (Additional file 2; Additional file 5). Whilst microbial community composition data is not available for all sponge species, both *C. foliascens* and *C. cyathophora* are known to host abundant populations of *Synechococcus* symbionts [13, 90, 91], and the presence of host thylakoid genes on viral contigs from other species suggests their microbiomes may also include *Synechococcus* symbionts. Enrichment of genes with assignment to this keyword shows that viruses could potentially interfere with photosynthetic processes in their hosts. Photosystem genes have also been observed in coral DNA viromes [45] and, with the exception of *C. marshae*, *P. candelabra* and *X. testudinaria*, were present in all sponge species investigated (Fig. 6).

Collagen genes were abundant within the sponge viromes, being present in all sponge species (Fig. 5), and a significant driver of functional differences between host species (Fig. 6) and sampling locations (Fig. 7). A previous study also identified collagen genes as being an abundant component of sponge viromes, and a key driver of functional differences between sponge, coral and seawater viromes [31]. Contigs containing genes encoding collagen proteins were consistently attributed to dsDNA viruses (Additional file 5), and when assigned at the family level, they included members of the bacteriophage families *Myoviridae*, *Podoviridae* and *Siphoviridae*, the algal *Phycodnaviridae*, the crustacean-infecting *Nimaviridae* and the giant virus family *Mimiviridae* (Additional file 5). Collagen is an integral structural component of the external capsid of members of the *Mimiviridae* [92] but is also used by sponges to form their skeletal structure [93]. Whilst it is clear that collagen genes are an important component of sponge-associated prokaryotic and eukaryotic viruses, their functional role within the sponge virome remains unclear and warrants further investigation.

Genes coding for ankyrin repeat proteins (ARPs) were only enriched in *C. schulzei* contigs. These ARPs likely originated from a member of the *Megavirales*, as the only family-level taxonomic assignments made to contigs containing ARPs belonged to the *Phycodnaviridae*, *Iridoviridae*, *Mimiviridae* or *Poxviridae* (Additional file 5). The ankyrin repeat is an amino-acid motif that can disrupt protein–protein interactions in cellular processes [94, 95]. *Herpesviridae* and *Poxviridae* have been previously shown to encode ARPs [96–98] and it has been suggested that they play a role in enhancing the adaptive capacity of the host via suppression of the cellular response to hypoxia [97], ubiquitination and immune responses [96]. Whilst most of the ARP genes in the *C. shulzei* viromes were assigned to *Megavirales*, contigs assigned to bacteriophage families also contained these proteins (Additional file 5). Recently, Jahn and colleagues showed that sponge bacteriophage can encode ankyrin domains that, upon expression in bacteria, reduce the eukaryotic immune response and subsequent phagocytosis of bacteria [36]. Horizontal gene transfer of ARPs amongst diverse symbionts has been proposed as a possible mechanism explaining their widespread distribution in sponges [99] and their enrichment in the virome of some sponge species indicates that this horizontal transfer may be viral mediated.

Arsenic, tellurium, chromate, molybdite and cadmium resistance genes were enriched in many viromes isolated from Red Sea sponges, and contigs containing these genes were assigned as bacteriophage within the order *Caudovirales* (Additional file 5). Some sponge species appear to have an exceptionally high tolerance for heavy metals and can bioaccumulate them from comparatively low concentrations in the surrounding environment. For instance, *Tedania charcoti* can accumulate and tolerate extraordinarily high concentrations of cadmium, even when environmental exposure is low [100]. In comparison to elevated levels of heavy metal contamination in the Red Sea, largely attributed to industrial and human activities in the coastal area [101], levels in the GBR are generally low, particularly within the GBR Marine Park [102]. Enrichment of these genes in Red Sea sponge viromes in comparison to their absence or comparatively low representation within GBR sponges, suggest that viruses may be contributing to heavy metal resistance in their host microbes.

Several genes from viromes isolated from Red Sea sponges had homology to known nylon degradation genes, and all originated from contigs assigned as *Caudovirales* (Additional file 5) [103]. Genes associated with nylon degradation have previously been characterised from Flavobacteria and Pseudomonas species [104–106]. Pollution from synthetic plastic compounds has increased considerably in marine ecosystems [107], and microplastics have recently been detected within the gastrointestinal tracts of Red Sea fish [103]. Enrichment of heavy metal and nylon degrading genes in Red Sea sponge viromes, together with previous reports of heavy metal and plastic contamination at the coastal sampling site, suggests that viruses could support host resistance to environmental contaminant exposure.

Pesticides and herbicides associated with agricultural runoff can occur at high levels in coastal and lagoonal areas of the GBR [108, 109]. Genes related to herbicide resistance were primarily assigned to contigs from *Synechococcus* phages, and these were supported by VirSorter analysis to be viral in origin (Additional file 5). *Synechococcus* is the most abundant cyanobacterium in the ocean and a major contributor to the productivity of coastal seawater [110]. The toxicological effects of herbicides on cyanobacterial populations is well documented [111–113]; hence, the presence of herbicide resistance genes in GBR sponges may provide a pathway for environmental acclimatisation of phototrophic species to agricultural runoff. Herbicide resistance genes identified within these samples shared homology to known Photosystem II proteins (Additional file 5), but further validation would be required to determine whether these genes are capable of conveying resistance to elevated environmental herbicide levels.

An enhanced potential for nitrogen metabolism is a key feature of the microbiome of most HMA sponge species [114]. Here, we detected a significant enrichment of the Swiss-Prot keywords ‘nitrate assimilation’ and ‘nitrogen fixation’ in the viromes of sponges containing high microbial abundance (HMA) (Additional file 3: Fig. S2). Largely attributed to viral contigs assigned to *Myoviridae* or *Phycodnaviridae*, genes associated with nitrate assimilation included nitrate and nitrite reductases and associated transport genes, and genes associated with nitrogen fixation included glutamine synthetases and a large number of nitrogen fixation and regulation proteins (Additional file 5). These results suggest that the virome may be contributing key genes involved in nitrogen metabolism in HMA sponges or that targeting the nitrogen metabolism pathway is part of the viral infection strategy in these species.

Genes assigned the ‘Antimicrobial’ keyword were enriched in LMA sponge species (Additional file 3: Fig. S2) and were largely limited to contigs taxonomically assigned as bacteriophage. Genes annotated with ‘antimicrobial’ activity were largely hydrolytic enzymes, with VirSorter support for viral taxonomic assignment (Additional file 5). Sponges are renowned for their production of bioactive secondary metabolites, although with a few notable exceptions [115], it is generally unknown whether these antimicrobial compounds originate from the host or the microbial symbionts. Hydrolytic enzymes with antimicrobial activities have previously been identified in a sponge microbial metagenome [116] and here we show a potential bacteriophage origin for at least some antimicrobial genes.

## Conclusion

Comparative analysis of viral communities from 15 sponge species collected from different geographic regions (GBR vs Red Sea), and representing different host nutritional modes (photosymbionts vs no photosymbionts) and strategies for microbial symbiosis (HMA vs LMA), has greatly expanded our understanding of viral ecology in marine sponges. dsDNA viruses spanning all three families of the *Caudovirales* as well as the NCLDV families *Mimiviridae*, *Marseilleviridae*, *Phycodnaviridae*, *Ascoviridae*, *Iridoviridae*, *Asfarviridae* and *Poxviridae* were present in sponges. ssDNA viruses from the *Microviridae*, *Circoviridae* and *Inoviridae*, as well as the *Retroviridae* were also prevalent, although their relative abundance was more variable across sponge species. Whilst core viral functions related to replication, infection and structure were consistent across most sponge species, functional profiles varied significantly between species and sites, in part attributed to differential representation of putative AMGs and accessory genes. Genes associated with herbicide resistance, heavy metal resistance and nylon degradation were differentially represented across sampling sites, whereas putative AMGs associated with antimicrobial activity were enriched in low microbial abundance species, nitrogen metabolism in high microbial abundance species and cellulose biosynthesis in sponge species hosting photosynthetic cyanobacteria (Fig. 8). These results highlight the diverse suite of beneficial roles viral putative AMGs and accessory genes may play in the functional ecology of the sponge holobiont.

## Methods

### Sample collection

As sampling criteria, we opted for collecting sponge species that represented the dominant sponge fauna [117–120] at two distinct biogeographical regions with varying levels of anthropogenic impact. Additionally, we selected sponges that presented different host nutritional modes (photosymbionts vs no photosymbionts) and strategies for microbial symbiosis (high microbial abundance—HMA vs low microbial abundance—LMA). Triplicate samples of nine coral reef sponges species—*Callyspongia* sp., *Carteriospongia foliascens*, *Cinachyrella schulzei*, *Cymbastella marshae*, *Echinochalina isaaci*, *Ianthella basta*, *Lamellodysidea herbacea*, *Pipestela candelabra*, *Stylissa carteri* were collected from Orpheus Island, Queensland, Australia (18° 35′ 34″ S, 146° 28′ 53″ E) and seven sponge species—*Amphimedon ochracea*, *Carteriospongia foliascens*, *Crella cyathophora*, *Hyrtios erectus*, *Mycale* sp., *Niphates rowi*, *Xestospongia testudinaria* were collected in Al Fahal, Saudi Arabia (22° 13′ 95″ N, 39° 01′ 81″ E), between December 2015 and February 2016 (Additional file 1). Sponges were collected on SCUBA between 3 and 15 m depth. All specimens were photographed in situ before being individually placed in sterile tubes and immediately transferred on ice to the laboratory for purification of viral particles. A seawater sample was collected from each sampling location (*n* = 1 × 20 l) as a comparative reference for the sponge samples. Seawater was collected using sterile containers and stored at 4 °C for 2-24 h prior to being filtered through a 0.22-μm Sterivex polyethersulfone filter.

### Viral concentration, purification and TEM analysis

Isolation and purification of sponge viruses were performed using a modified version of the protocol designed to isolate VLP from culture lysates and coral tissue [64, 121]. Approximately 25 g of fresh sponge tissue was cut into small pieces (5 mm), covered with 15 μl of 0.02 μm filter-sterilised (Whatman Anotop, Merck, Darmstadt, Germany) SM buffer (100 mM NaCl, 8 mM MgSO_4_, 50 mM Tris pH 7.5), and homogenised with a Craig’s HS30E homogeniser (Witeg, Wertheim, Germany) for 5 to 10 min. This step of virus purification involves the physical and chemical disruption of cells to release the viral particles. Tissue homogenates were filtered through a 100-μm cell strainer (Corning, New York, NY, USA) to eliminate sponge tissue, then centrifuged at 500 g for 15 min at 4 °C to pellet the cellular debris. Supernatant was subsequently transferred to a new sterile tube.

Supernatant density was brought to 1.2 g ml^−1^ with the addition of caesium chloride (CsCl). In parallel, different density CsCl solutions in 0.02 μm filtered SM buffer, were layered in the ultracentrifuge tube (3 ml of 1.6 g ml^−1^ solution; 2.5 ml of 1.45 g ml^−1^ solution; 2.5 ml of 1.3 g ml^−1^ solution; 2 ml of 1.2 g ml^−1^ solution). A 7.5-ml aliquot of each sponge sample brought to 1.2 g ml^−1^ cesuim chloride solution was dispensed on top of three gradient tubes (2.5 ml per tube), and centrifuged (Beckman Coulter Ultracentrifuge, Brea, CA, USA) in a swinging-bucket rotor (SW 40 Ti) for 2 h 40 min at 40,000 g, at 4 °C. Following centrifugation, the tube content was fractionated by density into eighteen fractions. The density and nucleic acid concentration of each fraction was determined [64], and the fractions with density between 1.2 g/ml and 1.5 g/ml were pooled together and filtered (0.22 μm EMD Millipore filter, Merck) to remove any remaining cellular contamination. Buffer exchange was performed to remove the CsCl salt from the samples by loading each sample into a 30 KDa Amicon centrifugal spin column (Millipore), centrifuging at 4000 g for 30 min at 4 °C, discarding the flow-through and repeating this operation four to six times to ensure complete exchange of CsCl into filter-sterilised SM buffer. A final centrifugation step resulted in the concentration of VLPs into a 600-μl solution of filter-sterilised SM buffer. In total, 200 μl of this solution was used for DNA extraction, whilst 100 μl was fixed in 0.5% glutaraldehyde for TEM analysis.

Viruses were purified from seawater using tangential flow filtration (30 kDa, Pall Corporation, New York, NY, USA) [122], by concentrating viruses from 20 l of pre-filtered (0.22 μm EMD Millipore filter) seawater into 20 ml seawater solution. Diafiltration was performed to replace seawater with SM buffer and samples were concentrated to a final volume of 500 μl using Amicon centrifugal spin columns (30 kDa, Millipore) as decribed above.

### Viral DNA extraction and amplification for sequencing

To degrade any free nucleic acid residing outside the viral capsid, purified viral samples were treated with DNase and RNase (Ambion, Thermo Fisher Scientific, Waltham, MA, USA) prior to DNA extraction according to the manufacturer’s instructions. DNA was extracted using the FastDNA SPIN Kit for Soil (MP Biomedicals, Santa Ana, CA, USA) following the manufacturer’s instructions. A modified random priming-mediated sequence-independent single-primer amplification (RP-SISPA) approach was used to amplify viral DNA fragments [64]. Briefly, viral DNA was converted to dsDNA using a Klenow fragment (3′–5′ exo-) using RP-SISPA primers with a 3′ random hexamer sequence. Eight microlitres of DNA was added to 6 μl of reaction mix containing 1.5 μl of 10× NEB buffer (New England Biolabs. Ipswich, MA, USA); 1 μl of 2.5 mM dNTPs; 1.5 μl of primer FR26RV-N (GCCGGAGCTCTGCAGATATCNNNNNN, 10 μM stock) and 2 μl of DNase-free distilled water. Reactions were incubated at 94 °C for 3 min, placed on ice for 3 min (primer annealing) before 1 μl of Klenow fragment was added to the mix and incubated at 37 °C for 60 min. After incubation, 1 μl of dNTP and 1 μl of N primer were add to each tube, samples were incubated at 94 °C for 3 min and placed on ice for 3 min. Lastly, 1 μl of Klenow was added to the solution and the reaction was incubated at 37 °C for 60 min then terminated at 75 °C for 20 min. Triplicate PCR amplifications were performed using the SISPA template. Two microlitres of template was added to 23 μl of reaction mix containing 2.5 μl of 10× reaction buffer, 4 μl of dNTP (2.5 mM stock), 2 μl of FR20RV primer (GCCGGAGCTCTGCAGATATC, 10μMstock) and 0.25 μl of TaKaRa LA HS Taq polymerase (5 U/μl, Scientifix, South Yarra, VIC, Australia). Reactions were incubated at 95 °C for 10 min, followed by 30 denaturation cycles (95 °C for 30 s, 60 °C for 60 s, 72 °C for 90 s) and a final hold at 72 °C for 13 min to enable completion of complementary strand synthesis. PCR reactions were loaded onto a 0.8% agarose gel in 1 × TAE at 100 V for 30 min. Amplifications with no visible PCR product were repeated by diluting the SISPA template 10 or 100 times. A reconditioning PCR was performed after pooling triplicate reactions to avoid sequencing artefacts [123]. Ten microlitres of pooled template was added in 90 μl of mix containing 55.25 μl of PCR water, 10 μl 10× reaction buffer, 16 μl dNTP (2.5 mM stock), 8 μl FR20RV primer (10μMstock) and 0.75 μl TaKaRa LA HS Taq. Reactions were incubated as per the PCR amplification protocol and cleaned using the MinElute PCR purification kit (Qiagen, Hilden, Germany). Samples were run on a 0.8% agarose gel in 1 × TAE at 100 V for 30 min and DNA quality (260:280 ratios) was assessed on a NanoDrop 2000 (Thermo Fisher Scientific).

### Viral DNA sequencing and bioinformatic analysis

All purified viral DNA was sequenced using TruSeq SBS kit V4 s125 bp fragments paired-end sequencing (Illumina) at the Bioscience Core Lab at the King Abdullah University of Science and Technology-KAUST, Thuwal, Saudi Arabia.

Sequence data was analysed based on the HoloVir protocol [30], a computational workflow designed for assigning taxonomy and function to host-associated viruses. The HoloVir protocol incorporates sequence quality evaluation, viral genomic assembly and gene prediction, together with taxonomic and functional analysis, whilst also undertaking an evaluation of cellular contamination in order to best characterise host-associated viral assemblages. However, we cannot eliminate the possibility of residual cellular contigs within viromic datasets. Quality control (QC) was performed on raw sequence data using CLC Genomic Workbench version 9.0 (Cambridge, MA, USA), where library adaptors, ambiguous nucleotides (*n* = 2) and low-quality bases (0.01) were trimmed, and reads below 40 bp were discarded. Viral metagenomes were assembled from the trimmed sequences using the de novo assembly function in CLC Genomic Workbench. Contigs smaller than 500 bp in length or with an average coverage value below 3 were discarded. Gene prediction was performed on the contigs using MetaGeneAnnotator [124]. Predicted genes were used for viral taxonomic assignment and functional annotation. Sequence abundance counts were calculated for each contig by mapping original input reads to assembled contigs using BWA. Contig fasta files were formatted to include MEGAN-compatable coverage values in the sequence descriptions [30]. Taxonomic assignment was performed using MEGAN6 [125], utilising BLAST analysis to search for homology between predicted gene data and the known viral reference genome within the NCBI RefSeq database [126]. MEGAN6 was run using a top-percent parameter of 80, min-support value of five reads and a bit score threshold value of 80. Assembled data was also compared to the HoloVir cellular and viral marker database to identify any cellular contamination [30]. Viral taxonomic classification was based on a lowest common ancestor scoring system using the best significant matches to viral reference sequences, and cellular contamination was evaluated using the HoloVir cellular and viral marker database to identify contigs of cellular origin. All contigs underwent additional evaluation using VirSorter, a tool designed to identifiy viral contigs within cellular metagenomic datasets [42]. VirSorter assignments are listed within Additional file 5, noting that VirSorter is typically unable to assess contigs less than 3000 bp in length.

Functional analysis of predicted genes was performed as described in the HoloVir protocol [30], utilising BLASTP sequence similarity searches of predicted genes against the Swiss-Prot manually curated UniprotKB protein database [127], using an *e–*value cutoff of 10^−10^, a cutoff range specifically chosen to capture and identify functional homology [128]. Swiss-Prot keywords were assigned to each predicted gene based on the best significant BLASTP match. Overall keyword frequency was calculated for each virome by adjusting for both contig coverage as well as keyword frequency within the Swiss-Prot database. Additional file 5 highlights all contigs from all datasets containing genes assigned specific functional keywords. Uniprot IDs for each gene are listed, together with the contig of origin, HoloVir taxonomic assignment, source contig length, the total number of taxonomic matches occurring on each contig and any VirSorter viral category that was assigned to the contigs. Detailed functional assignment was manually performed on the most frequent Swiss-Prot keyword categories based on literature review, and genes were assigned to four broad categories; genes involved in viral infection, replication, structural formation and auxillary genes (Fig. 5). All scripts, including R scripts and CLC assembly workflow files used in this study can be found on Github (https://github.com/AIMS/HoloVir). All R analyses was performed via R studio 1.3, and utilized R version 3.6.3, using R pagkages ggplot2, reshape2, vegan, pheatmap and mvabund.

### Data analyses

Permutational multivariate analysis of variance (PERMANOVA) was performed to identify significant differences in viral community composition and functional profiles between host species, sampling sites, host nutritional mode and microbial abundance. The PERMANOVA design considered ‘host species’ nested within ‘sampling site’. Additional pair-wise tests were conducted using 999 Monte Carlo permutations to determine significant differences amongst sponge species. Similarity percentage (SIMPER) analysis was used to identify viral taxa that contributed most to the dissimilarities identified by PERMANOVA. To visualise sample separation according to host features, non-metric multidimensional scaling (MDS) analyses were performed using Hellinger-transformed data. Analyses testing differences in viral community composition were performed using genus-level taxonomic assignments for predicted genes whilst functional differences were tested using Swiss-Prot functional keyword assignments for predicted genes, both normalised according to taxon or keyword frequency in the database. Multivariate analyses were performed on raw values based on Bray-Curtis dissimilarity matrix using Primer v 6.1.7 (PRIMER-E Ltd., Plymouth, UK) and univariate tests were performed using the R package mvabund (R version 3.6.3) [129] to identify functional drivers of differences (*P* value ≤ 0.02) between host sponge species (Fig. 6) and sampling location (Fig. 7).

### Transmission electron microscopy analysis

Virus-like particles (VLP) associated with the sponges were visualised and morphologically described using transmission electron microscopy and three different sample preparation methods: (i) sponge ultrathin sections [130]; (ii) viral purification via density gradient solution [64, 121]; (iii) sponge mucus scraping [131]. All samples were fixed in 1.5% glutaraldehyde and stained with 1% uranyl acetate on copper grids prior to visualisation. All samples were examined using a Titan Cubed TEM (Thermo Fisher Scientific) at the KAUST. TEM search time was standardised to 1 h/sample.

## Supplementary information

**Additional file 1.** Summary of sampling locations, accessing numbers, sequencing statistics and cellular contamination evaluation of virome datasets. N50 values for each dataset were calculated based on evaluation of unfiltered contigs.

**Additional file 2.** Summary of MEGAN6 genus-level taxonomic assignment of viral genes using BLAST+ comparisons to the viral refseq database.

**Additional file 3. **This file includes: **Figure S1.** Non-metric multidimensional scaling plot based on Bray-Curtis similarity of Swiss-Prot functional keyword for predicted genes. **Figure S2.** Heatmap of viral functions that were significantly different between HMA and LMA sponges. **Figure S3.** Heatmap of viral functions that were significantly different between sponges with and without photosymbionts. **Table S1.** Pairwise PERMANOVA results of predicted viral genes associated with fifteen sponge species from the Great Barrier Reef and the Red Sea based on viral RefSeq taxonomic assignments (genus level). **Table S2.** Average similarity (%) between and within sponge species from the Great Barrier Reef based on viral RefSeq gene taxonomic assignments (genus level). **Table S3.** Average similarity (%) between and within sponge species from the Red Sea based on Swiss-Prot keyword abundance data. **Table S4.** Pairwise PERMANOVA results of predicted viral genes associated with fifteen sponge species from the Great Barrier Reef and the Red Sea. **Table S5.** Average similarity (%) between and within sponge species from the Great Barrier Reef based Swiss-Prot keyword abundance data. **Table S6.** Average similarity (%) between and within sponge species from the Red Sea based on Swiss-Prot keyword abundance data.

**Additional file 4.** Similarity percentage analysis (SIMPER) of differences in viral metagenomes associated with sponges with and without photosymbionts, based on Bray-Curtis similarity of genus-level taxonomic assignment of predicted genes. The columns two and three identify the average abundance of viral taxa within each sponge group, the fourth and fifth columns represent the Bray Curtis dissimilarity metric between sponges with and without photosymbionts and its standard deviation, respectively. The sixth column shows the percentage of dissimilarity contribution explained by that viral taxa and the seventh column shows the cumulative Bray Curtis dissimilarity metric for the taxa. The eighth and ninth columns represent the natural hosts for the viral group according the International Committee on Taxonomy of Viruses (ICTV).

**Additional file 5.** ## 1 - Complete phage contigs - category 1 (sure)

## Abbreviations

AMGs: Auxiliary metabolic genes
TEM: Transmission electron microscopy
VLP: Viral-like particles
HMA: High microbial abundance
LMA: Low microbial abundance
RP-SISPA: Random priming-mediated sequence-independent single-primer amplification
MDS: Non-metric multidimensional scaling
QC: Quality control
