# Supplementary material for: Viral ecogenomics across the Porifera
Source: Microbiome. 2020 Oct 2;8:144. doi: 10.1186/s40168-020-00919-5 (PMC7532657; doi:10.1186/s40168-020-00919-5)
Supplement: Supplementary file 4 — Additional file 3. This file includes: Figure S1. Non-metric multidimensional scaling plot based on Bray-Curtis similarity of Swiss-Prot functional keyword for predicted genes. Figure S2. Heatmap of viral functions that were significantly different between HMA and LMA sponges. Figure S3. Heatmap of viral functions that were significantly different between sponges with and without photosymbionts. Table S1. Pairwise PERMANOVA results of predicted viral genes associated with fifteen sponge species from the Great Barrier Reef and the Red Sea based on viral RefSeq taxonomic assignments (genus level). Table S2. Average similarity (%) between and within sponge species from the Great Barrier Reef based on viral RefSeq gene taxonomic assignments (genus level). Table S3. Average similarity (%) between and within sponge species from the Red Sea based on Swiss-Prot keyword abundance data. Table S4. Pairwise PERMANOVA results of predicted viral genes associated with fifteen sponge species from the Great Barrier Reef and the Red Sea. Table S5. Average similarity (%) between and within sponge species from the Great Barrier Reef based Swiss-Prot keyword abundance data. Table S6. Average similarity (%) between and within sponge species from the Red Sea based on Swiss-Prot keyword abundance data. [file 40168_2020_919_MOESM3_ESM.docx]

Additional file 3 for

**Viral ecogenomics across the Porifera**

Running Header: Reef Sponge Viromics

Cecília Pascelli#,1,2,3, Patrick W. Laffy#,2, Emmanuelle Botté2, Marija Kupresanin4, Thomas Rattei5, Miguel Lurgi6, Timothy Ravasi4 and Nicole S. Webster*1,2,7

Correspondence: * [n.webster@aims.gov.au](mailto:ceciliapascelli@gmail.com)

^#^ Joint first Authors

This file includes:

**Figure S1.** Non-metric multidimensional scaling plot based on Bray-Curtis similarity of Swiss-Prot functional keyword for predicted genes.

**Figure S2.** Heatmap of viral function that were significantly different between HMA and LMA sponges.

**Figure S3.** Heatmap of viral functions that were significantly different between sponges with and without photosymbionts.

**Table S1.** Pairwise PERMANOVA results of predicted viral genes associated with fifteen sponge species from the Great Barrier Reef and the Red Sea based on viral RefSeq taxonomic assignments (genus level).

**Table S2.** Average similarity (%) between and within sponge species from the Great Barrier Reef based on viral RefSeq gene taxonomic assignments (genus level).

**Table S3**. Average similarity (%) between and within sponge species from the Red Sea based on Swiss-Prot keyword abundance data.

**Table S4.** Pairwise PERMANOVA results of predicted viral genes associated with fifteen sponge species from the Great Barrier Reef and the Red Sea.

**Table S5.** Average similarity (%) between and within sponge species from the Great Barrier Reef based Swiss-Prot keyword abundance data.

**Table S6.** Average similarity (%) between and within sponge species from the Red Sea based on Swiss-Prot keyword abundance data.


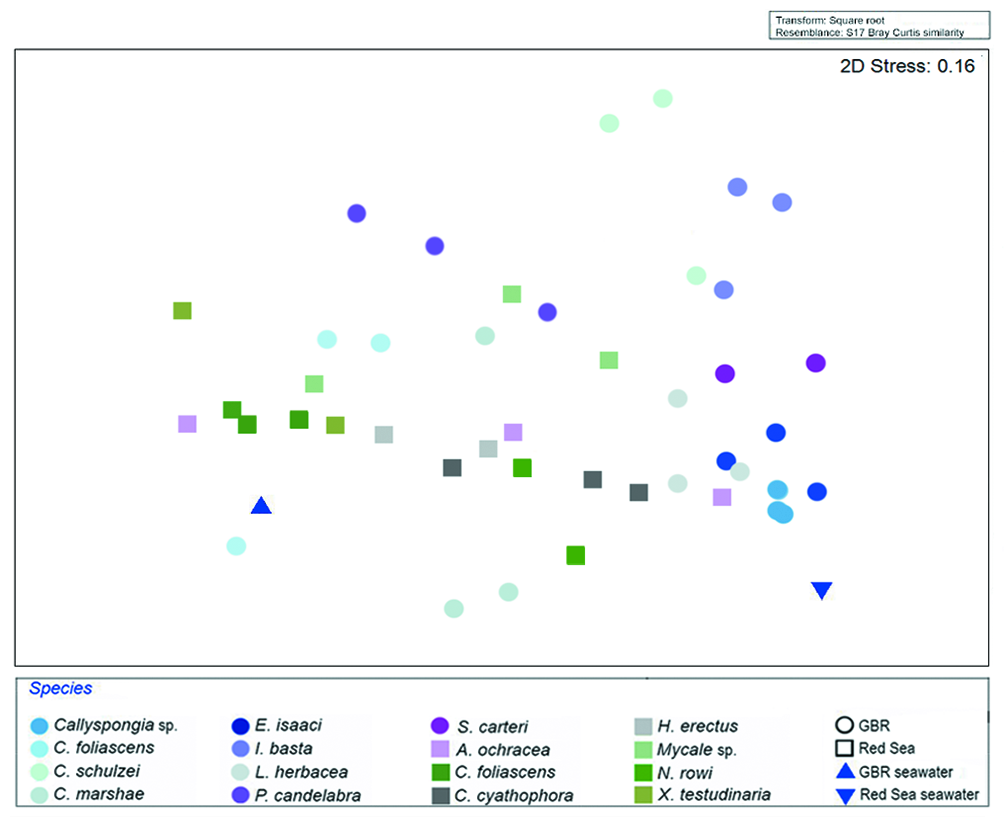


**Figure S1. Non-metric multidimensional scaling plot based on Bray Curtis similarity of Swiss-Prot functional keyword for predicted genes.** Ordination displays similarities in the viral communities of the fifteen sponge species from the Great Barrier Reef and the Red Sea.


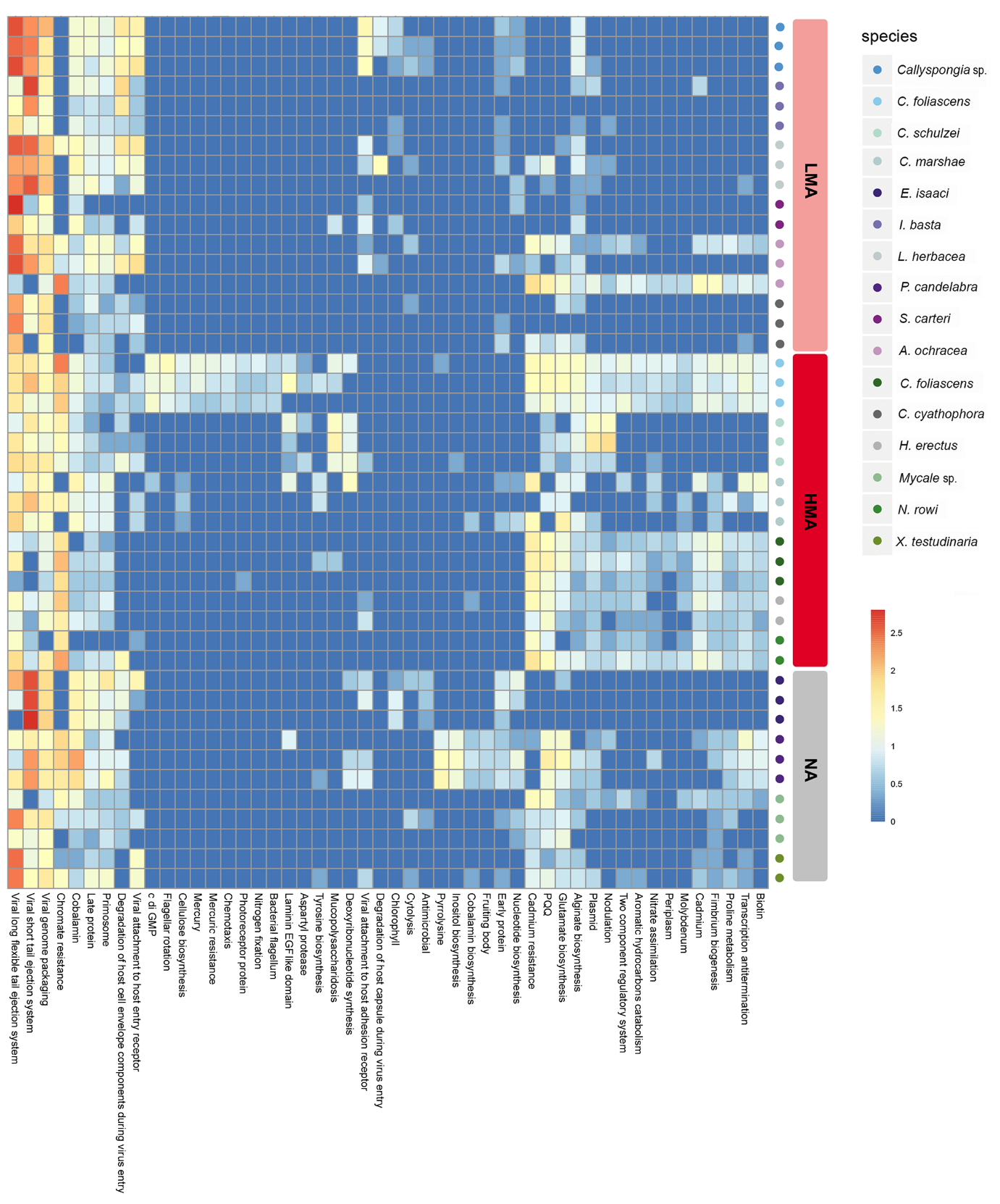


**Figure S2. Heatmap of viral function that were significantly different between HMA and LMA sponges.** Mvabund was used to perform univariate tests on Swiss-Prot keywords abundance data, identifying key drivers of differences between HMA and LMA sponges. Heatmap shows significant differences between Swissprot Keyword enrichment frequency data from all samples (P value <0.02), adjusted to account for coverage of the source contig within individual viromes.


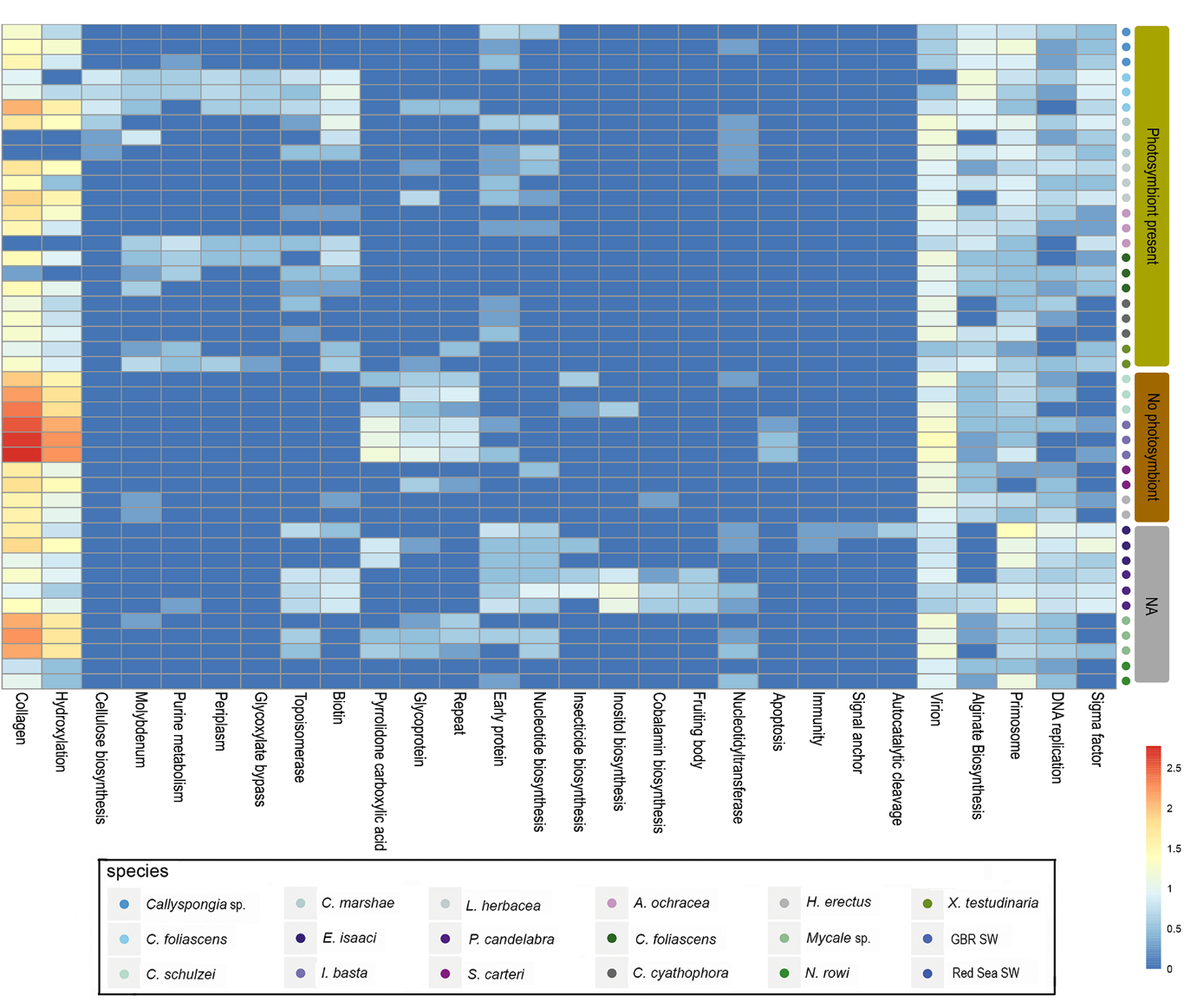


**Figure S3. Heatmap of viral functions that were significantly different between sponges with and without photosymbionts.** Mvabund was used to perform univariate tests on Swiss-Prot keyword abundance data, identifying key drivers of viromic functional differences between photosymbiont-hosting and non-hosting sponges. Heatmap shows significant differences between Swissprot Keyword enrichment frequency data from all samples (P value <0.02), adjusted to account for coverage of the source contig within individual viromes.

**Table S1. Pairwise PERMANOVA results of predicted viral genes associated with fifteen sponge species from the Great Barrier Reef and the Red Sea based on viral RefSeq taxonomic assignments (genus level).** Multivariate permutational analysis of variance (PERMANOVA) (Primer6/PERMANOVA+ v1.0.2, Plymouth, UK) was used to compare taxonomic differences in the viral communities amongst replicates from different sponges within each site. Samples with significant p-values (≤0.05) by Monte Carlo (MC) tests and permutation tests are highlighted with an asterix (*). The degrees of freedom are indicated by the letters “df”, the Student’s t-test are indicated by the letter “t”; “Unique perms” indicates how many unique-values of the test statistic were obtained under permutation.

| Site | Sponge species | df | t | P (MC) | P(perm) | Unique perms |
| --- | --- | --- | --- | --- | --- | --- |
| Great Barrier Reef | *Callyspongia* sp*., C. foliascens* | 4,4 | 38,834 | 0,006* | 0,111 | 10 |
|  | *Callyspongia* sp.*, C. schulzei* | 4,4 | 26,536 | 0,018* | 0,104 | 10 |
|  | *Callyspongia* sp.*, C. marshae* | 4,4 | 19,943 | 0,036* | 0,119 | 10 |
|  | *Callyspongia* sp.*, E. isaaci* | 4,4 | 20,851 | 0,034* | 0,103 | 10 |
|  | *Callyspongia* sp.*, I. basta* | 4,4 | 31,266 | 0,006* | 0,086 | 10 |
|  | *Callyspongia* sp.*, L. herbacea* | 4,4 | 1,792 | 0,081 | 0,098 | 10 |
|  | *Callyspongia* sp.*, P. candelabra* | 4,4 | 37,906 | 0,005* | 0,105 | 10 |
|  | *Callyspongia* sp.*, S. carteri* | 4,3 | 22,529 | 0,055 | 0,097 | 10 |
|  | *Callyspongia* sp.*, Sea water - GBR* | 4,2 | 49,722 | 0,02* | 0,263 | 4 |
|  | *C. foliascens, C. schulzei* | 4,4 | 23,071 | 0,016* | 0,089 | 10 |
|  | *C. foliascens, C. marshae* | 4,4 | 19,853 | 0,025* | 0,098 | 10 |
|  | *C. foliascens, E. isaaci* | 4,4 | 38,198 | 0,003* | 0,095 | 10 |
|  | *C. foliascens, I. basta* | 4,4 | 37,121 | 0,002* | 0,099 | 10 |
|  | *C. foliascens, L. herbacea* | 4,4 | 21,938 | 0,02* | 0,101 | 10 |
|  | *C. foliascens, P. candelabra* | 4,4 | 25,771 | 0,021* | 0,109 | 10 |
|  | *C. foliascens, S. carteri* | 4,3 | 24,166 | 0,028* | 0,105 | 10 |
|  | *C. foliascens, Sea water - GBR* | 4,2 | 16,014 | 0,176 | 0,246 | 4 |
|  | *C. schulzei, C. marshae* | 4,4 | 1,686 | 0,066 | 0,082 | 10 |
|  | *C. schulzei, E. isaaci* | 4,4 | 24,548 | 0,017* | 0,112 | 10 |
|  | *C. schulzei, I. basta* | 4,4 | 22,833 | 0,03* | 0,104 | 10 |
|  | *C. schulzei, L. herbacea* | 4,4 | 1,951 | 0,045* | 0,103 | 10 |
|  | *C. schulzei, P. candelabra* | 4,4 | 21,508 | 0,032* | 0,092 | 10 |
|  | *C. schulzei, S. carteri* | 4,3 | 17,712 | 0,102 | 0,086 | 10 |
|  | *C. schulzei, Sea water - GBR* | 4,2 | 16,391 | 0,164 | 0,234 | 4 |
|  | *C. marshae, E. isaaci* | 4,4 | 21,106 | 0,022* | 0,109 | 10 |
|  | *C. marshae, I. basta* | 4,4 | 19,199 | 0,039* | 0,08 | 10 |
|  | *C. marshae, L. herbacea* | 4,4 | 14,007 | 0,141 | 0,117 | 10 |
|  | *C. marshae, P. candelabra* | 4,4 | 14,513 | 0,121 | 0,119 | 10 |
|  | *C. marshae, S. carteri* | 4,3 | 14,485 | 0,141 | 0,115 | 10 |
|  | *C. marshae, Sea water - GBR* | 4,2 | 12,718 | 0,275 | 0,252 | 4 |
|  | *E. isaaci, I. basta* | 4,4 | 22,664 | 0,021* | 0,097 | 10 |
|  | *E. isaaci, L. herbacea* | 4,4 | 20,607 | 0,025* | 0,101 | 10 |
|  | *E. isaaci, P. candelabra* | 4,4 | 32,027 | 0,012* | 0,081 | 10 |
|  | *E. isaaci, S. carteri* | 4,3 | 2,231 | 0,039* | 0,1 | 10 |
|  | *E. isaaci, Sea water - GBR* | 4,2 | 29,881 | 0,057 | 0,262 | 4 |
|  | *I. basta, L. herbacea* | 4,4 | 2,306 | 0,013* | 0,114 | 10 |
|  | *I. basta, P. candelabra* | 4,4 | 31,998 | 0,009* | 0,098 | 10 |
|  | *I. basta, S. carteri* | 4,3 | 21,985 | 0,059* | 0,103 | 10 |
|  | *I. basta, Sea water - GBR* | 4,2 | 29,336 | 0,046* | 0,231 | 4 |
|  | *L. herbacea, P. candelabra* | 4,4 | 21,595 | 0,029* | 0,086 | 10 |
|  | *L. herbacea, S. carteri* | 4,3 | 17,958 | 0,076 | 0,089 | 10 |
|  | *L. herbacea, Sea water - GBR* | 4,2 | 19,632 | 0,091 | 0,239 | 4 |
|  | *P. candelabra, S. carteri* | 4,3 | 24,418 | 0,038* | 0,109 | 10 |
|  | *P. candelabra, Sea water - GBR* | 4,2 | 23,295 | 0,079 | 0,253 | 4 |
|  | *S. carteri, Sea water - GBR* | 3,2 | 13,852 | 0,355 | 0,326 | 3 |
| Red Sea | *C. foliascens, A. ochracea* | 4,4 | 12,827 | 0,241 | 0,302 | 10 |
|  | *C. foliascens, C. cyathophora* | 4,4 | 3,01 | 0,006* | 0,112 | 10 |
|  | *C. foliascens, H. erectus* | 4,3 | 2,429 | 0,044* | 0,091 | 10 |
|  | *C. foliascens, Mycale* sp. | 4,4 | 22,269 | 0,038* | 0,097 | 10 |
|  | *C. foliascens, N. rowi* | 4,3 | 31,526 | 0,013* | 0,114 | 10 |
|  | *C. foliascens, X. testudinaria* | 4,3 | 14,777 | 0,164 | 0,091 | 10 |
|  | *C. foliascens, RS_SW* | 4,2 | 39,668 | 0,024* | 0,266 | 4 |
|  | *A. ochracea, C. cyathophora* | 4,4 | 11,607 | 0,266 | 0,207 | 10 |
|  | *A. ochracea, H. erectus* | 4,3 | 0,7921 | 0,577 | 0,716 | 10 |
|  | *A. ochracea, Mycale* sp. | 4,4 | 0,78289 | 0,611 | 0,698 | 10 |
|  | *A. ochracea, N. rowi* | 4,3 | 0,90854 | 0,478 | 0,801 | 10 |
|  | *A. ochracea, X. testudinaria* | 4,3 | 10,702 | 0,379 | 0,274 | 10 |
|  | *A. ochracea, Sea water - RS* | 4,2 | 11,669 | 0,333 | 0,509 | 4 |
|  | *C. cyathophora, H. erectus* | 4,3 | 18,306 | 0,071 | 0,096 | 10 |
|  | *C. cyathophora, Mycale* sp. | 4,4 | 1,175 | 0,285 | 0,193 | 10 |
|  | *C. cyathophora, N. rowi* | 4,3 | 14,456 | 0,162 | 0,194 | 10 |
|  | *C. cyathophora, X. testudinaria* | 4,3 | 24,785 | 0,034* | 0,094 | 10 |
|  | *C. cyathophora, Sea water - RS* | 4,2 | 17,362 | 0,13 | 0,264 | 4 |
|  | *H. erectus, Mycale* sp. | 3,4 | 1,101 | 0,335 | 0,284 | 10 |
|  | *H. erectus, N. rowi* | 3,3 | 16,815 | 0,17 | 0,327 | 3 |
|  | *H. erectus, X. testudinaria* | 3,3 | 20,895 | 0,112 | 0,358 | 3 |
|  | *H. erectus, Sea water - RS* | 3,2 | 30,172 | 0,138 | 0,34 | 3 |
|  | *Mycale* sp. *N. rowi* | 4,3 | 12,263 | 0,272 | 0,196 | 10 |
|  | *Mycale* sp.*, X. testudinaria* | 4,3 | 17,873 | 0,109 | 0,212 | 10 |
|  | *Mycale* sp.*, Sea water - RS* | 4,2 | 16,268 | 0,174 | 0,241 | 4 |
|  | *N. rowi, X. testudinaria* | 3,3 | 25,491 | 0,077 | 0,341 | 3 |
|  | *N. rowi, Sea water - RS* | 3,2 | 1,615 | 0,298 | 0,318 | 3 |
|  | *X. testudinaria, Sea water – RS* | 3,2 | 30,559 | 0,149 | 0,332 | 3 |

**Table S2. Average similarity (%) between and within sponge species from the Great Barrier Reef based on viral RefSeq gene taxonomic assignments (genus level).**

|  | *Callyspongia* sp. | *C. foliascens* | *C. schulzei* | *C. marshae* | *E. isaaci* | *I. basta* | *L. herbacea* | *P. candelabra* | *S. carteri* | Sea water GBR |
| --- | --- | --- | --- | --- | --- | --- | --- | --- | --- | --- |
| *Callyspongia* sp. | 91.7 |  |  |  |  |  |  |  |  |  |
| *C. foliascens* | 65.0 | 81.0 |  |  |  |  |  |  |  |  |
| *C. schulzei* | 64.5 | 63.0 | 72.0 |  |  |  |  |  |  |  |
| *C. marshae* | 72.2 | 67.5 | 65.6 | 73.4 |  |  |  |  |  |  |
| *E. isaaci* | 81.2 | 58.3 | 62.7 | 67.7 | 83.8 |  |  |  |  |  |
| *I. basta* | 74.7 | 59.5 | 64.6 | 69.9 | 75.1 | 83.9 |  |  |  |  |
| *L. herbacea* | 80.1 | 70.8 | 66.7 | 73.1 | 73.9 | 71.8 | 80.3 |  |  |  |
| *P. candelabra* | 71.2 | 70.7 | 66.8 | 74.6 | 68.1 | 68.3 | 73.6 | 84.7 |  |  |
| *S. carteri* | 67.9 | 58.3 | 60.0 | 66.1 | 63.2 | 63.8 | 66.2 | 61.2 | 68.7 |  |
| Sea water GBR | 65.8 | 72.7 | 59.1 | 68.5 | 59.1 | 60.3 | 66.2 | 69.4 | 59.4 | 0.0 |

**Table S3. Average similarity (%) between and within sponge species from the Red Sea based on viral RefSeq gene taxonomic assignments (genus level).**

|  | *C. foliascens* | *A. ochracea* | *C. cyathophora* | *H. erectus* | *Mycale* sp. | *N. rowi* | *X. testudinaria* | Seawater RS |
| --- | --- | --- | --- | --- | --- | --- | --- | --- |
| *C. foliascens* | 86.2 |  |  |  |  |  |  |  |
| *A. ochracea* | 72.9 | 66.7 |  |  |  |  |  |  |
| *C. cyathophora* | 64.7 | 70.3 | 78.0 |  |  |  |  |  |
| *H. erectus* | 76.8 | 74.9 | 72.9 | 86.9 |  |  |  |  |
| *Mycale* sp. | 70.9 | 73.2 | 76.0 | 78.9 | 76.6 |  |  |  |
| *N. rowi* | 67.1 | 73.4 | 75.4 | 78.9 | 76.7 | 82.3 |  |  |
| *X. testudinaria* | 81.3 | 71.2 | 63.6 | 75.1 | 70.7 | 66.2 | 82.2 |  |
| Seawater RS | 54.8 | 63.4 | 66.2 | 65.3 | 65.8 | 73.8 | 52.3 | 0.0 |

**Table S4. Pairwise PERMANOVA results of predicted viral genes associated with fifteen sponge species from the Great Barrier Reef and the Red Sea based on Swiss-Prot keyword abundance data.** Multivariate permutational analysis of variance (PERMANOVA) (Primer6/PERMANOVA+ v1.0.2, Plymouth, UK) was used to compare taxonomic differences in the viral communities amongst replicates from different sponges within each site. Samples with significant p-values (≤0.05) by Monte Carlo (MC) tests and permutation tests are highlighted with an asterix (*). The degrees of freedom are indicated by the letters “df”, the Student’s t-test are indicated by the letter “t”; “Unique perms” indicates how many unique-values of the test statistic were obtained under permutation.

| Site | Sponge species | df | t | P (MC) | P(perm) | Unique perms |
| --- | --- | --- | --- | --- | --- | --- |
| Great Barrier Reef | Callyspongia sp., C. foliascens | 4,4 | 5.0278 | 0.003* | 0.095 | 10 |
|  | Callyspongia sp., C. schulzei | 4,4 | 2.7455 | 0.013* | 0.1 | 10 |
|  | Callyspongia sp., C. marshae | 4,4 | 2.7784 | 0.013* | 0.103 | 10 |
|  | Callyspongia sp., E. isaaci | 4,4 | 2.6161 | 0.013* | 0.101 | 10 |
|  | Callyspongia sp., I. basta | 4,4 | 4.847 | 0.003* | 0.097 | 10 |
|  | Callyspongia sp., L. herbacea | 4,4 | 1.6889 | 0.096 | 0.102 | 10 |
|  | Callyspongia sp., P. candelabra | 4,4 | 4.0858 | 0.004* | 0.096 | 10 |
|  | Callyspongia sp., S. carteri | 4,3 | 2.1914 | 0.054 | 0.097 | 10 |
|  | Callyspongia sp., Sea water GBR | 4,2 | 8.1287 | 0.006* | 0.257 | 4 |
|  | C. foliascens, C. schulzei | 4,4 | 2.4584 | 0.016* | 0.079 | 10 |
|  | C. foliascens, C. marshae | 4,4 | 1.954 | 0.036* | 0.085 | 10 |
|  | C. foliascens, E. isaaci | 4,4 | 4.2431 | 0.003* | 0.097 | 10 |
|  | C. foliascens, I. basta | 4,4 | 3.855 | 0.004* | 0.109 | 10 |
|  | C. foliascens, L. herbacea | 4,4 | 3.1602 | 0.007* | 0.09 | 10 |
|  | C. foliascens, P. candelabra | 4,4 | 2.5167 | 0.011* | 0.094 | 10 |
|  | C. foliascens, S. carteri | 4,3 | 2.6203 | 0.028* | 0.1 | 10 |
|  | C. foliascens, Sea water GBR | 4,2 | 1.3097 | 0.25 | 0.274 | 4 |
|  | C. schulzei, C. marshae | 4,4 | 1.8619 | 0.034* | 0.094 | 10 |
|  | C. schulzei, E. isaaci | 4,4 | 2.4226 | 0.019* | 0.11 | 10 |
|  | C. schulzei, I. basta | 4,4 | 1.9991 | 0.036* | 0.105 | 10 |
|  | C. schulzei, L. herbacea | 4,4 | 2.0019 | 0.037* | 0.104 | 10 |
|  | C. schulzei, P. candelabra | 4,4 | 2.077 | 0.03* | 0.078 | 10 |
|  | C. schulzei, S. carteri | 4,3 | 1.4157 | 0.175 | 0.204 | 10 |
|  | C. schulzei, Sea water GBR | 4,2 | 1.6522 | 0.167 | 0.25 | 4 |
|  | C. marshae, E. isaaci | 4,4 | 2.3578 | 0.022* | 0.105 | 10 |
|  | C. marshae, I. basta | 4,4 | 2.6311 | 0.013* | 0.1 | 10 |
|  | C. marshae, L. herbacea | 4,4 | 1.8782 | 0.044* | 0.096 | 10 |
|  | C. marshae, P. candelabra | 4,4 | 1.4078 | 0.14 | 0.093 | 10 |
|  | C. marshae, S. carteri | 4,3 | 1.5628 | 0.122 | 0.11 | 10 |
|  | C. marshae, Sea water GBR | 4,2 | 1.3043 | 0.274 | 0.231 | 4 |
|  | E. isaaci, I. basta | 4,4 | 3.7963 | 0.005* | 0.109 | 10 |
|  | E. isaaci, L. herbacea | 4,4 | 1.5414 | 0.103 | 0.107 | 10 |
|  | E. isaaci, P. candelabra | 4,4 | 3.353 | 0.009* | 0.092 | 10 |
|  | E. isaaci, S. carteri | 4,3 | 1.9125 | 0.069 | 0.106 | 10 |
|  | E. isaaci, Sea water GBR | 4,2 | 4.0656 | 0.022* | 0.255 | 4 |
|  | I. basta, L. herbacea | 4,4 | 2.9348 | 0.01* | 0.096 | 10 |
|  | I. basta, P. candelabra | 4,4 | 3.4078 | 0.005* | 0.11 | 10 |
|  | I. basta, S. carteri | 4,3 | 2.1351 | 0.046* | 0.085 | 10 |
|  | I. basta, Sea water GBR | 4,2 | 3.2505 | 0.032* | 0.221 | 4 |
|  | L. herbacea, P. candelabra | 4,4 | 2.6588 | 0.012* | 0.111 | 10 |
|  | L. herbacea, S. carteri | 4,3 | 1.3916 | 0.182 | 0.097 | 10 |
|  | L. herbacea, Sea water GBR | 4,2 | 2.4595 | 0.058 | 0.242 | 4 |
|  | P. candelabra, S. carteri | 4,3 | 2.2799 | 0.03* | 0.089 | 10 |
|  | P. candelabra, Sea water GBR | 4,2 | 1.9325 | 0.095 | 0.255 | 4 |
|  | S. carteri, Sea water GBR | 3,2 | 1.8486 | 0.239 | 0.347 | 3 |
| Red Sea | C. foliascens, A. ochracea | 4,4 | 1.3604 | 0.213 | 0.397 | 10 |
|  | C. foliascens, C. cyathophora | 4,4 | 3.0452 | 0.006* | 0.116 | 10 |
|  | C. foliascens, H. erectus | 4,3 | 1.7734 | 0.107 | 0.104 | 10 |
|  | C. foliascens, Mycale sp. | 4,4 | 2.0104 | 0.063 | 0.082 | 10 |
|  | C. foliascens, N. rowi | 4,3 | 3.3022 | 0.012* | 0.103 | 10 |
|  | C. foliascens, X. testudinaria | 4,3 | 1.4469 | 0.164 | 0.103 | 10 |
|  | C. foliascens, Sea water RS | 4,2 | 5.2429 | 0.019* | 0.264 | 4 |
|  | A. ochracea, C. cyathophora | 4,4 | 1.187 | 0.292 | 0.204 | 10 |
|  | A. ochracea, H. erectus | 4,3 | 0.61285 | 0.675 | 0.712 | 10 |
|  | A. ochracea, Mycale sp. | 4,4 | 1.0188 | 0.402 | 0.389 | 10 |
|  | A. ochracea, N. rowi | 4,3 | 0.74434 | 0.616 | 0.715 | 10 |
|  | A. ochracea, X. testudinaria | 4,3 | 1.0605 | 0.378 | 0.41 | 10 |
|  | A. ochracea, Sea water RS | 4,2 | 1.1428 | 0.379 | 0.481 | 4 |
|  | C. cyathophora, H. erectus | 4,3 | 1.8516 | 0.077 | 0.111 | 10 |
|  | C. cyathophora, Mycale sp. | 4,4 | 1.4398 | 0.146 | 0.102 | 10 |
|  | C. cyathophora, N. rowi | 4,3 | 1.5066 | 0.144 | 0.109 | 10 |
|  | C. cyathophora, X. testudinaria | 4,3 | 2.1882 | 0.046* | 0.103 | 10 |
|  | C. cyathophora, Sea water RS | 4,2 | 1.7739 | 0.142 | 0.238 | 4 |
|  | H. erectus, Mycale sp. | 3,4 | 1.2093 | 0.284 | 0.333 | 10 |
|  | H. erectus, N. rowi | 3,3 | 1.5681 | 0.218 | 0.323 | 3 |
|  | H. erectus, X. testudinaria | 3,3 | 1.3298 | 0.276 | 0.329 | 3 |
|  | H. erectus, Sea water RS | 3,2 | 2.7631 | 0.164 | 0.342 | 3 |
|  | Mycale sp., N. rowi | 4,3 | 1.5352 | 0.142 | 0.111 | 10 |
|  | Mycale sp., X. testudinaria | 4,3 | 1.4438 | 0.177 | 0.202 | 10 |
|  | Mycale sp., Sea water RS | 4,2 | 1.8174 | 0.133 | 0.264 | 4 |
|  | N. rowi, X. testudinaria | 3,3 | 2.0868 | 0.101 | 0.347 | 3 |
|  | N. rowi, Sea water RS | 3,2 | 1.5629 | 0.307 | 0.326 | 3 |
|  | X. testudinaria, Sea water RS | 3,2 | 2.5781 | 0.177 | 0.318 | 3 |

**Table S5. Average similarity (%) between and within sponge species from the Great Barrier Reef based Swiss-Prot keyword abundance data.**

|  | *Callyspongia* sp. | *C. foliascens* | *C. schulzei* | *C. marshae* | *E. isaaci* | *I. basta* | *L. herbacea* | *P. candelabra* | | *S. carteri* | | Sea water GBR |
| --- | --- | --- | --- | --- | --- | --- | --- | --- | --- | --- | --- | --- |
| *Callyspongia* sp. | 88.9 |  |  |  |  |  |  |  |  | |  | |
| *C. foliascens* | 28.6 | 68.7 |  |  |  |  |  |  |  | |  | |
| *C. schulzei* | 39.0 | 34.4 | 52.7 |  |  |  |  |  |  | |  | |
| *C. marshae* | 38.2 | 44.2 | 36.2 | 52.8 |  |  |  |  |  | |  | |
| *E. isaaci* | 69.9 | 29.7 | 40.4 | 41.5 | 78.1 |  |  |  |  | |  | |
| *I. basta* | 42.6 | 32.0 | 46.4 | 34.6 | 44.3 | 74.7 |  |  |  | |  | |
| *L. herbacea* | 69.8 | 36.5 | 43.1 | 45.2 | 66.8 | 45.7 | 68.0 |  |  | |  | |
| *P. candelabra* | 37.2 | 45.7 | 40.3 | 52.7 | 40.3 | 36.0 | 42.9 | 66.1 |  | |  | |
| *S. carteri* | 56.3 | 33.2 | 45.7 | 42.0 | 54.5 | 48.1 | 57.0 | 37.9 | 56.7 | |  | |
| Sea water GBR | 25.2 | 61.8 | 30.5 | 42.7 | 25.5 | 30.6 | 33.0 | 43.0 | 27.3 | | 0.0 | |

**Table S6. Average similarity (%) between and within sponge species from the Red Sea based on** **Swiss-Prot keyword abundance data.**

|  | *C. foliascens* | *A. ochracea* | *C. cyathophora* | *H. erectus* | *Mycale* sp. | *N. rowi* | *X. testudinaria* | Seawater RS |
| --- | --- | --- | --- | --- | --- | --- | --- | --- |
| *C. foliascens* | 81.6 |  |  |  |  |  |  |  |
| *A. ochracea* | 58.9 | 47.2 |  |  |  |  |  |  |
| *C. cyathophora* | 45.1 | 51.9 | 64.3 |  |  |  |  |  |
| *H. erectus* | 71.8 | 63.1 | 54.0 | 74.0 |  |  |  |  |
| *Mycale* sp. | 54.2 | 51.4 | 53.2 | 58.9 | 56.4 |  |  |  |
| *N. rowi* | 49.9 | 60.0 | 59.2 | 65.4 | 51.3 | 71.7 |  |  |
| *X. testudinaria* | 70.6 | 53.7 | 43.5 | 65.3 | 51.8 | 48.8 | 65.0 |  |
| Seawater RS | 20.2 | 43.6 | 44.6 | 36.7 | 29.5 | 59.8 | 20.1 | 0.0 |
